# 25th Annual Meeting of the Rocky Mountain Virology Association

**DOI:** 10.3390/v18040464

**Published:** 2026-04-14

**Authors:** Talia J. Byrne-Haber, Kylee N. Pham, Arianna Joob, Samantha M. Pinto, Oshani C. Ratnayake, Ryan Thompson, Joel Rovnak, Rushika Perera

**Affiliations:** Department of Microbiology, Immunology, and Pathology, College of Veterinary Medicine and Biomedical Sciences, Colorado State University, Fort Collins, CO 80523, USA; taliabh@colostate.edu (T.J.B.-H.); kylee.pham@colostate.edu (K.N.P.); arianna.joob@colostate.edu (A.J.); samantha.pinto@colostate.edu (S.M.P.); oshani.ratnayake@colostate.edu (O.C.R.); ryan.thompson@colostate.edu (R.T.); joel.rovnak@colostate.edu (J.R.)

**Keywords:** virus, prions, immunology, infection, transmission, structural biology, vector biology

## Abstract

Located on the traditional and ancestral homelands of the Arapaho, Cheyenne, and Ute Nations, Colorado State University’s Mountain Campus hosted the 25th Annual Rocky Mountain Virology Association meeting. The three-day event, held from 26 September to 28 September 2025, welcomed 152 participants focused on the following topics: viruses, prions, immunology, transmission, structural biology, and vector biology. This year’s Randall Jay Cohrs Keynote Presentation summarized ongoing research on viral glycoproteins in relation to viral entry and assembly. Understanding the role of viral glycoproteins is essential in vaccine and antiviral development for enveloped RNA viruses. Alongside rigorous scientific discourse and networking, attendees made the most of their time by hiking amidst beautiful fall colors, wildlife, and young aspens starting the forest anew. On behalf of the Rocky Mountain Virology Association, this report summarizes select presentations from the 25th annual meeting.

## 1. Introduction

**Figure 1:**
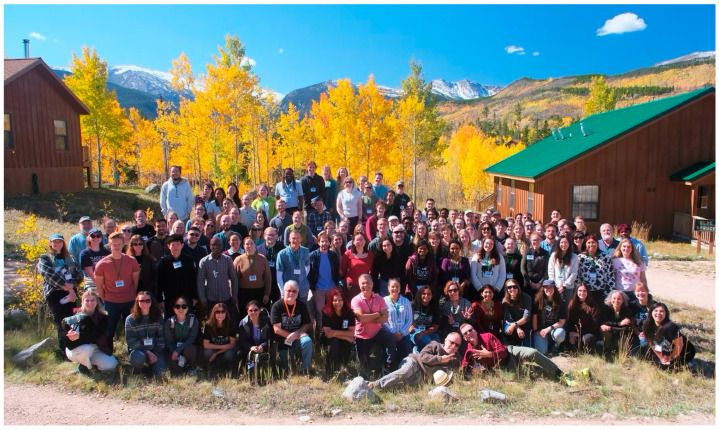
Attendees of the 25th Rocky Mountain Virology Association meeting. Photo taken by Tony Schountz.

**Figure 2:**
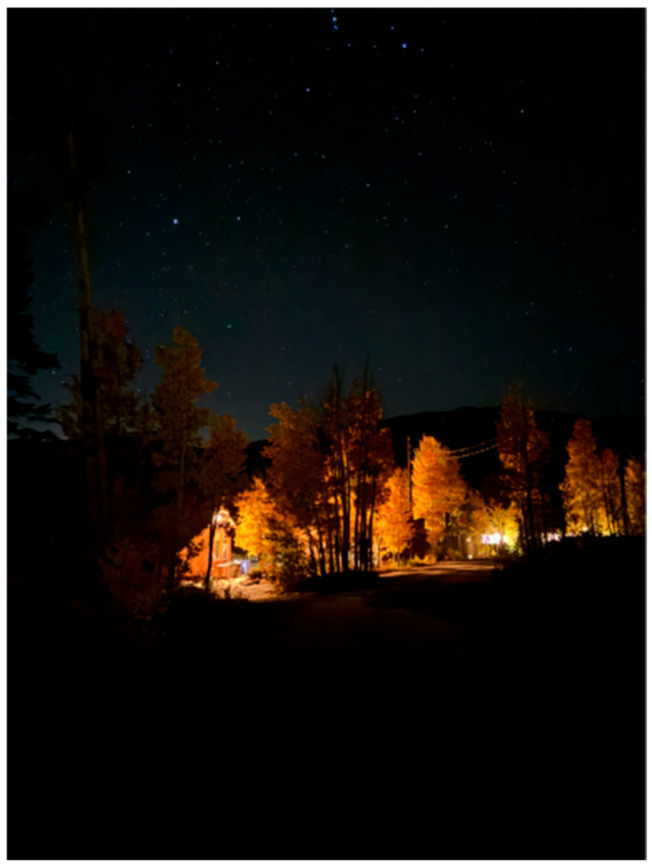
Fall colors illuminated by the night sky at the CSU Mountain Campus. Photo taken by Kylee Pham.

Just 50 miles from Fort Collins, Colorado State University’s Mountain Campus is the home to the Rocky Mountain Virology Association annual meeting. Founded in 2000, the Rocky Mountain Virology Club (RMVC) opened its doors to virologists at any stage in their careers, providing them a place to share their expertise and collaborate with others. In 2009, RMVC expanded to include prion biologists. The following year, RMVC was renamed the Rocky Mountain Virology Association (RMVA). Like the few aspens emerging from burn scars to begin a new forest, what started as a meeting of a handful of people has grown dramatically over the last 25 years. Attendees gathered at Colorado State University’s Mountain Campus from 26 September to 29 September 2025 (Figure 1 and Figure 2). The conference featured five sessions, six invited speakers, 33 oral presentations, and 57 poster presentations.

The meeting started with the Randall Jay Cohrs Keynote Presentation delivered by Dr. Hector Aguilar-Carreno from the University of California, Los Angeles’ Department of Microbiology, Immunology, and Molecular Genetics. He presented on enveloped RNA virus glycoproteins and their roles in viral entry and assembly, and how this research can be used for vaccine and antiviral development.

The Richard A. Bessen Memorial Lecture was delivered by Dr. David Harris from Boston University. Dr. Harris spoke on elacridar’s effects on lysosomal sterol and lipid metabolism in relation to the scrapie isoform of the prion protein. This year’s national and international invited speakers included Dr. Patrick Dolan from the National Institute of Allergy and Infectious Diseases, who discussed viral capsids and environmental stressors, Dr. Priscilla Yang from Stanford University, who spoke on small molecule inhibitors against flaviviruses, Dr. Andrew Bubak from University of Colorado Anschutz, who presented on Varicella Zoster Virus and neurodegenerative disease, and Dr. Kei Sato from the University of Tokyo, who delivered remarks on the evolution of sarbecoviruses. This year’s meeting also included an industry session featuring Dr. Timothy Tellinghuisen from Roche and Dr. Alexander Ball from GeneTex.

To engage the audience, poster presenters gave lightning talks. These informal, delightful presentations of three minutes or less consisted of sing-along songs, a DJ, a prion spin-off of *Family Feud*, and storytelling. Like always, the RMVA Annual Meeting showcased virology, prion biology, teamwork, fun, and mentorship. Selected abstracts are presented below.

## 2. Summary of Scientific Sessions

### 2.1. The Randall Jay Cohrs Lecture––Keynote Address by Dr. Hector Aguilar-Carreno

Dr. Hector Aguilar-Carreno from the Department of Microbiology, Immunology, and Molecular Genetics at the University of California, Los Angeles, presented his lab’s research on elucidation of key components in viral glycoproteins in their hosts that (1) mediate viral entry into mammalian cells, (2) elicit immune responses, and (3) mediate viral assembly and egress from cells. The lab focuses on basic research in viral entry and assembly, as well as preclinical research in vaccine and antiviral development for enveloped RNA viruses, with the strongest emphasis on paramyxoviruses such as the deadly henipaviruses, but also significant emphasis on orthomyxoviruses, coronaviruses, and pneumoviruses. They have developed several new quantitative and kinetic techniques to dissect the mechanistic steps by which glycoproteins modulate viral entry. Further, using viral-like particles and pseudotyped virions, they developed highly neutralizing conformational monoclonal antibodies that block various specific steps in the viral entry process, as well as unique techniques to analyze and characterize such viral particles and the host immune responses they elicit. They study both the viral and cellular factors involved in these processes, and these studies are yielding novel ways to develop broad-spectrum vaccines and antivirals. All animal studies were performed following guidelines and protocols approved by the Institutional Animal Care and Use Committee of the University of California, Los Angeles.

### 2.2. Current Affairs and Novel Tools in Virology

Adam Catching, in collaboration with Walker Symonds-Orr and Patrick T. Dolan from the Quantitative Virology and Evolution Unit, Laboratory of Viral Diseases, NIH-NIAID Division of Intramural Research, Bethesda, MD, presented their research using mutational scanning to understand how viral capsids sense their environment. Viral capsids are remarkable molecular machines. They have evolved to be robust to the environmental stresses encountered, which often include changes in pH, salinity, and temperature, while maintaining precise mechanisms to release their genomic cargo when specific criteria are met—usually when receptors and co-receptors are presented within the right chemical environment. Here, they use a mutational library encompassing all possible substitutions in the capsid proteins of Enterovirus D68 and Enterovirus A71 to probe capsid functions involved in entry and uncoating. The results highlight specific regions that respond to chemical and biophysical changes and how mutational preferences shift in response, and identify functional sequences that may engage host factors to initiate and facilitate uncoating and entry. This research was supported by the Intramural Research Program of the National Institutes of Health (NIH) under Project 1ZIAAI001360. The contributions of the NIH authors were made as part of their official duties as NIH federal employees, follow agency policy requirements, and are considered Works of the United States Government. However, the findings and conclusions presented in this paper are those of the authors and do not necessarily reflect the views of the NIH or the U.S. Department of Health and Human Services.

Taylor Crisologo, Mary Nehring, Treana Mayer, Sachithra Gunasekara, Craig Miller, Jennifer Rudd, Angela Bosco-Lauth, Tyler Sherman, Christie Mayo, and Sue VandeWoude from Colorado State University, College of Veterinary Medicine and Biomedical Sciences, Department of Microbiology, Immunology, and Pathology, Fort Collins, CO, and Oklahoma State University, College of Veterinary Medicine, Department of Veterinary Pathobiology, Stillwater, OK, discussed their efforts studying the broadly cross-reactive antibodies detected in cats infected with SARS-CoV-2 variants WA-1 and Omicron XBB.1.5 in an enzyme-linked immunosorbent assay (ELISA). Since its initial isolation and characterization in December 2019, SARS-CoV-2 has evolved into several important variant lineages. The prototype genome was isolated in Wuhan, China, and is closely related to the first variant isolated in the U.S. (Washington State, WA-1) in January 2020. Characterized in November 2021, the Omicron variant (B.1.1.529) resulted in a high number of human cases compared to other variants and is still in circulation. Experimental infection of cats with SARS-CoV-2 has shown they are susceptible to infection with multiple variants and capable of transmission to conspecifics by direct contact. Crisologo developed ELISAs to detect antibodies against Omicron spike (S1 + S2) and receptor-binding domain (RBD) antigens for comparison to existing WA-1 ELISAs to further our understanding of Omicron seroprevalence in domestic cats. They detected cross-reactivity of antibodies from cats experimentally infected with Omicron XBB.1.5 at 4–5 days post-inoculation at titers of 1:4000 against Omicron B.1.1.529 and >1:9000 against Wuhan-Hu-1-derived viral spike antigens. Seroreactivity against the RBD was less robust, particularly for Omicron RBD. They further assessed cross-reactivity of cats infected with the WA-1 strain against Omicron B.1.1.529 RBD and spike antigens and noted high seroreactivity from samples collected approximately 6 weeks post-infection in all assays. Neutralizing antibodies against Wuhan-Hu-1 and Omicron B.1.1.529 variants were also detected in WA-1-infected cats in a surrogate virus neutralization test. Their results indicate that domestic cats can mount a robust and broadly cross-reactive antibody response to multiple SARS-CoV-2 variants as early as 4–5 days post-infection, and that Omicron may be less immunogenic than Wuhan-Hu-1 SARS-CoV-2 isolates in cats. Future studies will assess seroprevalence and cross-reactivity in domestic cats from samples submitted to Colorado State University Veterinary Diagnostic Laboratory between 2021 and 2023. They will also assess potential risk factors such as sex, age, breed, and comorbidities. All animal studies were performed following guidelines and protocols approved by the Institutional Animal Care and Use Committee of Colorado State University. This project was funded in part by a Young Investigator Grant from the Center for Companion Animal Studies and Colorado State University’s Veterinary Summer Scholars Program.

Juliette Dashe, working with Trevor Hale, Yazeed Aljohani, Shelby Cagle, and Nicole Kelp from the Department of Statistics, College of Natural Sciences, College of Veterinary Medicine and Biomedical Sciences, Department of Ecosystem Science and Sustainability, Warner College of Natural Resources, Department of Microbiology, Immunology, and Pathology, College of Veterinary and Biomedical Sciences, Colorado State University, discussed their studies involving public perceptions of bats in emerging infectious disease research before and after COVID-19. Bats are an integral part of the ecosystem, serving as natural pest controllers, pollinators, and seed dispersers. They are also recognized as reservoirs for zoonotic diseases, making them important laboratory models for studying emerging infections. Public scrutiny surrounding bat research escalated during and after the COVID-19 pandemic. Therefore, the research investigates public perceptions of emerging infectious disease (EID) research that utilizes bats as animal models, comparing narratives before and after COVID-19 and with research using other animal models. Public posts and comments were collected from Facebook, X, and Instagram via Google Social Search. After removing duplicates, 139 relevant posts from Colorado, Montana, Kansas, and Texas were analyzed across pre-, during, and post-COVID periods. Content analysis was conducted using MAXQDA, coding for themes such as zoonotic spillover, biosafety, and trust. Narratives of government and institutional distrust, along with frustration over uncertainty, outweighed expressions of ecological appreciation for bat-related research post-COVID. Colorado was the most prevalent state in discussions, largely due to scrutiny surrounding the new bat vivarium at Colorado State University. This research suggests that narratives of government distrust and misinformation around bat research increased during and after COVID. These findings can inform public health messaging strategies that prioritize transparency, build trust, and leverage local voices to correct misconceptions regarding EID research and bats as laboratory models. This research was funded by the National Institute of Allergy and Infectious Diseases of the National Institutes of Health under Award Number T32AI162691, and the CSU Office of the Vice President for Research, Thematic Unit of Excellence, Infectious Disease Research and Response Network. No animal or human studies were performed.

William B. Foreman from the University of Utah’s Department of Biochemistry, in collaboration with Bin Hu and Tyler N. Starr from the Department of Biochemistry at the University of Utah, discussed their efforts in designing pan-sarbecovirus monoclonal antibodies using machine learning and deep mutational scanning. Monoclonal antibodies that broadly neutralize across SARS-CoV-2 variants and the sarbecovirus lineage remain elusive, despite the existential threat to national security imposed by emerging sarbecoviruses. Broadly neutralizing antibodies to diverse sarbecoviruses are a tool for both treating and preventing infections with novel sarbecoviruses and SARS-CoV-2 variants. They aim to engineer a set of promising parental antibodies to achieve both breadth of binding and neutralization potency across the sarbecovirus lineage, properties that have so far been found to be mutually exclusive. To this aim, they have surveyed the mutational space of these parental antibodies for affinity towards a large sampling of sarbecoviruses using yeast surface display and deep mutational scanning with antibody mutant libraries. Using this mutational data, they are developing machine learning models to enhance the depth of sequence space that we can explore within the numerical confines of our library schemes, identifying affinity-enhancing mutations from these large datasets that elicit breadth of binding to multiple sarbecoviruses. They have successfully fine-tuned a general protein language model to achieve pan-sarbecovirus mutational prediction. Affinity-matured antibodies identified by these models will be expressed and characterized for breadth of binding to sarbecoviruses and vulnerability to escape by viral mutations using yeast surface display, assessed for neutralization potency, and computationally modeled to gain insight into mechanistic changes that occurred during affinity maturation. Understanding the tradeoff between breadth and neutralization potency in anti-sarbecovirus antibodies will inform the development of pan-sarbecovirus drug and vaccine design, alleviate the public health burden of SARS-CoV-2 and its variants, and potentially lead to a therapeutic that can protect against future sarbecovirus-related pandemics. No animals or human studies were performed.

Elizabeth Fortunato, Man I Kuan, Emmanuel C. Ijezie, and John M. O’Dowd from the Department of Biological Sciences at the University of Idaho discussed their studies exploring new ways to use our 3D Schwann cell/motor neuron co-culture system to study HCMV-induced late-onset sensori-neural hearing loss. Human Cytomegalovirus (HCMV) is a leading cause of congenital birth defects. The most common sequela observed in symptomatic infants is sensorineural hearing loss (SNHL). SNHL can also develop over a period of years in children born hearing-impaired and asymptomatic for infection. The most abundant peripheral nervous system (PNS) myelin protein, myelin protein zero (MPZ), is largely responsible for compaction of the PNS myelin sheath. They find MPZ expression dramatically decreased in congenitally HCMV-infected tissue samples. Schwann cells (SCs), the only cells that produce MPZ, support permissive, long-term HCMV infection, releasing a very low level of virions. HCMV infection or sole expression of exceedingly low levels of tegument protein pp71 in culture causes large decreases in MPZ mRNA levels. Since SCs only express MPZ protein when directly contacting the neurons they sheathe, they developed an all-human system to study the species-restricted human CMV pathogenesis. Preliminary experiments revealed the power of this system, showing robust neurite outgrowth, SC/neuron interactions, and MPZ expression in WT cocultures. They even observed myelin sheaths (via TEM) and the production of action potentials following electrical stimulation in these cocultures. Initial experiments with pp71-expressing SCs produced drastically decreased outgrowth, very different interactions between the cell types, little to no MPZ, and no evidence of myelination compared to wildtype. They have now begun to methodically quantify these differences and to explore various methods and timing of introduction of pp71, be it via inducible constructs, exosome exposure, or full infection, in an attempt to determine if timing and length of exposure influence the ability of the cocultures to function properly. Preliminary results from infection of SCs prior to coculture appear to more dramatically affect the examined parameters compared to sole pp71 expression. Their results may, for the first time, identify a causative pathway for the development of late-onset SNHL. This research was funded by NIAID, grant number R01AI139503 from the National Institutes of Health. No animal or human studies were performed.

Caleb Huntington from the University of California, Davis, working with Cassandra Bonavita, Isamara Navarette-Macias, Jay Tiemann, Heather Wells, and Simon Anthony, presented their research on environmental air sampling for wildlife viral surveillance. Wildlife viral surveillance is essential for understanding the pre-emergent pool of potential zoonoses. However, traditional surveillance methods face major challenges due to the need for large-scale efforts to detect rare and intermittently shed viruses. New technologies are needed to improve the discovery and characterization of unknown viral diversity. One such technology is environmental air sampling, which has a long history in detecting specific pathogens in indoor and agricultural settings but has yet to be systematically applied in a metagenomic capacity to wildlife viral surveillance. In this study, they investigate the ability of environmental air sampling to detect mammalian viruses in a cave-roosting bat assemblage. During the summers of 2023 and 2024, they conducted 115 air-sampling events in a cave in Puerto Rico where over 100,000 bats of six different species roost year-round. Air samples were screened for target viral families using a combination of next-generation sequencing and consensus PCR assays. They found that environmental air sampling successfully detected 14 mammalian viral families, including RNA and DNA viruses. The detection of both alpha and beta coronavirus highlights a critical use case for air sampling in bat populations. Additionally, air sampling conditions (flow rate, sampling duration, sampling location, time of day, and post-sample processing) were optimized to maximize viral abundance and diversity in the samples. The results showed that larger air sample volumes and sampling inside the cave while the bats were roosting had significant positive effects on overall detection. Their findings suggest that air sampling can recover diverse viral families as a non-invasive means of monitoring wildlife viruses. Future work will refine this approach to enhance viral discovery, transmission monitoring, and ecological surveillance. The research was made possible through a collaboration with the United States Department of Agriculture (USDA). All animal studies were performed following guidelines and protocols approved by the Institutional Animal Care and Use Committee of the University of California, Davis.

Victoria Klimuk, together with Treana Mayer, Michelle Galvan, and Sue Vandewoude from the Department of Microbiology, Immunology, and Pathology at Colorado State University, discussed their studies developing a nanopore-based sequencing protocol for feline immunodeficiency virus (FIV) whole-genome analysis. Feline immunodeficiency virus is challenging to study due to high mutation rates and variable clinical pathogenicity. Differences in viral replication, cell tropism, and apoptosis induction among FIV isolates suggest that genomic variation affects pathogenicity. However, clinical differences between subtypes are unclear. The seven subtypes identified are based on the *env* gene only, with several classified recombinant strains. Significant genetic variability among strains challenges the sensitivity of molecular diagnostic tests, resulting in unreliable PCR diagnostics and few published full-length FIV genomes. A next-generation sequencing tool from Oxford Nanopore Technologies could address these gaps with cost-efficient long-read sequences. Their project aims to develop the first ONT sequencing protocol for FIV to rapidly and accurately characterize FIV genomes. Furthermore, these methodologies could have applications in understanding other closely related retroviruses like the Human Immunodeficiency Virus. They explored current commercially available library preparation and sequencing workflows offered by ONT for applicability to FIV sequencing. A summary of comparisons includes introduced bias, expected quality and precision, time and resources, and RNA vs. DNA sequencing protocols. Workflows were evaluated under different FIV sequencing priorities and applications (e.g., whole genome vs. subtyping). Their results suggest minION direct RNA and cDNA sequencing protocols are best suited to this purpose, with the latter using a strand-switching method prior to PCR amplification (ONT protocol cDNA-PCR Sequencing V14 (SQK-PCS114). They will evaluate the accuracy, precision, and efficiency of these candidate workflows using a highly virulent FIV viral stock and clinical samples from a prior anti-retroviral therapy study. By obtaining full-length viral genomes from various timepoints during infection, they will investigate within-host and between-host viral evolution in experimentally infected individuals. This sequencing approach could ultimately provide novel insights into the genetic basis of FIV disease severity, including the risk of oncogenesis and the rapidity of onset of immunodeficiency. No animal or human studies were performed.

Thomas Tipih from National Institute of Allergy and Infectious Diseases (NIAID), working alongside Vignesh Mariappan, Kwe C Yinda, Kimberly Meade-White, Matthew Lewis, Atsushi Okumura, Natalie McCarthy, Kate Altynova, Shanna S. Leventhal, Trenton Bushmaker, Chad S Clancy, Emmie de Wit, Vincent J Munster, Heinz Feldmann, and Kyle Rosenke from the Laboratory of Virology at NIAID and Rocky Mountain Veterinary Branch at NIAID, detailed their findings of Highly Pathogenic Avian Influenza H5N1 clade 2.3.4.4b genotype B3.13 being highly virulent for mice, rapidly causing acute pulmonary and neurologic disease. The Highly Pathogenic Avian Influenza (HPAI) A(H5N1) clade 2.3.4.4b viruses, responsible for the current outbreak in dairy cows in the United States, present a major shift in our understanding of influenza A virus host range and pathogenesis. There are many gaps to fill, including countermeasure development for which animal models are critical. In this study, they compared disease progression and pathology of three recent clade 2.3.4.4b isolates derived from a cow, a mountain lion, and a mink to a human HPAI A(H5N1) isolate from Vietnam in mice. Inoculating C57BL/6J and BALB/c mice with all four HPAI A(H5N1) isolates resulted in comparable levels of virus replication in the lung, inducing significant local pro-inflammatory cytokine responses and severe respiratory disease. Infecting C57BL/6J mice with the bovine isolate yielded high viral titers in the brain, a significant pro-inflammatory cytokine response, and neurologic disease. Cow isolate replication in BALB/c mice was largely restricted to the lungs. Their findings suggest the bovine isolate possesses enhanced neuroinvasive/neurovirulent disease-causing fatal respiratory and neurologic disease in C57BL/6J mice. The C57BL/6J model allows studies on mechanisms of neuroinvasion and neurovirulence of the emerging HPAI A(H5N1) viruses. This work was funded by the Intramural Research Program of the National Institute of Allergy and Infectious Diseases (NIAID). Animal experiments were approved by the Rocky Mountain Laboratories Animal Care and Use Committee (ACUC) (protocol number 2024-19) and carried out in an AALAC-accredited facility.

Delaney Worthington from Colorado State University, in collaboration with Josh Hill, Jessica Gray, Alan Schenkel, and Nicole Kelp from the Department of Microbiology, Immunology, and Pathology at Colorado State University, presented their research on evaluating two approaches to science communication training in undergraduate virology. Integrating science communication into undergraduate education has become a growing priority across STEM. This need is especially prevalent in virology, where students must grapple with complex and evolving socioscientific issues like emerging infectious diseases (EIDs) and vaccine hesitancy. To prepare for careers in which they may have to communicate about topics like vaccine development, virus–host interactions, and viral evolution, students must develop the capacity to engage inclusively and intentionally with audiences beyond the scientific community. To this aim, they developed two science communication training interventions in an upper-division virology course. The first was a brief, 50 min module on inclusive science communication (ISC) and vaccine communication, integrated into a large, four-credit lecture-based virology course. The second intervention was a one-credit, semester-long group study for honors students who were also enrolled in the main, lecture-based section. Both interventions focused on training students in ISC practices. The 50 min module introduced ISC, strategic communication, health belief model, and included practice scenarios that applied to communicating about vaccines. Alternatively, the semester-long honors group study engaged students with ISC, scientific uncertainty, and EIDs through primary literature, class discussions, and interactive lectures. The honors students also created community-centered deliverables on a current viral EID and presented them in a community setting, giving them experience with creating virology-focused communication materials for diverse audiences. They collected quantitative data using the Planned Behaviors in Inclusive Science Communication (PB-ISC) scale and qualitative data from student reflections, which will be analyzed via thematic analysis. Here, they present the design frameworks of our trainings and preliminary results from our mixed-methods study, highlighting the impacts of these science communication interventions and modeling how science communication can be integrated into virology courses. This research was funded by the Teaching Innovation and Professional Development Grant from the Microbiology, Immunology, and Pathology Department at Colorado State University. All studies using human subjects or tissue samples have been either approved or deemed non-human-subject research by the Institutional Review Board of Colorado State University.

Priscilla Yang from Microbiology and Immunology at Stanford University conducted a research study on small-molecule-induced proximity as an antiviral strategy. Conventional direct-acting antivirals (DAAs) bind to and inhibit (or derange) the function of their viral targets. Their pharmacology is driven by target binding to the viral protein (occupancy), and any loss of binding due to point mutations reduces antiviral potency. This underlies the narrow-spectrum activity and propensity for resistance to DAAs used as monotherapies. In addition, it is difficult to design inhibitors of viral proteins that are not enzymes. Instead of using small-molecule binding to inhibit viral proteins, Yang’s group has been developing small molecules that achieve antiviral activity by inducing the proximity of the viral protein target with another protein. One example of this is small-molecule “degraders,” which bind to their viral target of interest and to a host E3 ubiquitin ligase. By inducing proximity between these two proteins, the small molecule can cause ubiquitination and subsequent degradation of the viral protein by the proteasome. The pharmacology is driven by the removal of the viral protein from the cell—once the protein has been ubiquitinated, no further binding to the viral protein is required. They hypothesized that this type of mechanism could have advantages in developing broad-spectrum activity, raising barriers to drug resistance, and providing an easier way to achieve antiviral activity against non-enzymatic viral proteins. They will describe their group’s proof-of-concept work developing antiviral degraders and comparing the ability of these compounds to address viral genetic diversity when compared to functional inhibitors. Research was funded by NIAID R01 AI146152 and R01 AI148632. No animals or human studies were performed.

### 2.3. Developments in Prion Research

Zoe Atkinson and Mary Hall, working together with Joseph P. DeFranco, Sehun Kim, Jenna Crowell, and Glenn C. Telling, presented their research on how the accumulation of thermolysin-resistant PrP correlates to neuropathology in a spontaneous disease model for Gerstmann–Sträussler–Scheinker syndrome. GSS is an inherited, sporadic disease linked to several point mutations in the human prion protein (PrP). GSS in humans results in ataxia, postural abnormalities, and cognitive decline, as well as amyloid plaque formation, lesioning, and other pathologies of the central nervous system (CNS). Neurological clinical signs and similar pathology in the CNS, as well as the abundance of Proteinase K (PK) resistant material, are hallmark characteristics of conventional prion diseases. Uniquely, GSS does not produce PK-resistant PrP, thus making it difficult to investigate with typical methods. To examine this syndrome more effectively, the Telling Lab created a transgenic mouse model that encodes a GSS-specific PrP point mutation that results in spontaneous neurological disease, herein known as Tg(GSS) mice. The objective of this study was to determine if there was a biochemical procedure that could detect GSS prions similar to the use of PK for conventional prions. When they assessed the progression of disease in Tg(GSS) mice, they found distinctive plaque and lesion profiles in the CNS that temporally correlate to the presence of Thermolysin-resistant PrP. Moreover, the occurrence of neuropathology in Tg(GSS) mice and the accumulation of Thermolysin-resistant PrP coincided with the decline of sensorimotor function. These results continue to broaden our understanding of GSS as an unconventional prion disease with undiscovered qualities. Useful models for studying spontaneous disease have historically been limited, raising challenges for the development of therapeutics for GSS and other prion diseases linked to PrP point mutations. The use of their model allows for the investigation of GSS in cell-free systems as well as the ability to assess future therapeutics as they apply to human disease. All animal studies were performed following guidelines and protocols approved by the Institutional Animal Care and Use Committee of Colorado State University. All studies using human subjects or tissue samples have been either approved or deemed non-human-subject research by the Institutional Review Board of Colorado State University.

Emily Gasowski, working with Kaitlyn Forrest and Candace K Mathiason, conducting research at Colorado State University’s Department of Microbiology, Immunology, and Pathology (Fort Collins, Colorado), presented research on the association between prion protein and circadian rhythms in transgenic mice. The prion protein (PrPC; gene Prnp) is expressed in all mammalian cells, yet to date, no definitive function has been described. One of several proposed roles for the prion protein involves its association with circadian rhythms. Yet few molecular studies have been performed to assess this hypothesis. Circadian rhythms are a form of temporal regulation in biological functions occurring within a 24 h cycle. Molecularly, a family of circadian transcription factors drives the expression of these rhythms as well as the expression of downstream targets that impact cellular function. Interestingly, the casein kinase 2 (CK2) protein is known to interact independently with both the molecular clock and the prion protein. The purpose of this study is to characterize molecular circadian rhythms in transgenic mice expressing varied levels of the prion protein (C57bl6/j (wildtype 1x expression), FVB-KO (Prnp knockout 0x expression), and Tg (CerPrP-E226 5037 (Prnp 4- 8x expression). The levels of Prnp and molecular clock genes (Clock, Bmal1, Period (1,2), Cryptochrome (1,2), Rev-erb (a,b)) were analyzed for each transgenic mouse line by qPCR. Additionally, Casein Kinase II alpha (Ck2a), Casein Kinase II Beta (Ck2b), and Prnp and molecular clock gene expression were analyzed by Western blot. Continued investigation of circadian rhythms in mammalian hosts is necessary to fully understand the correlation between prion protein expression in various tissues and its impact on the circadian clock. This work will help provide an understanding of functional losses in molecular circadian regulation due to the progression of prion disease. All animal studies were performed following guidelines and protocols approved by the Institutional Animal Care and Use Committee of Colorado State University.

David A Harris ^1^, researching together with Robert C.C. Mercer, Nhat T.T. Le ^1^, Nadia A. Mirza-Romero ^1^, Erin Flaherty ^2^, Joseph P. DeFranco ^2^, Giada Lavigna ^3^, Isabel C. Orbe ^1^, Jean R. P. Gatdula ^1^, Douglas G. Fraser ^4^, Aravind Sundaravadivelu ^1^, Janelle S. Vultaggio ^1^, Aaron B. Beeler ^4^, Roberto Chiesa ^3^, and Glenn C. Telling ^2^ (Boston University Chobanian and Avedisian School of Medicine, Department of Biochemistry and Cell Biology, Boston, Massachusetts, USA, ^2^ Department of Microbiology, Immunology, and Pathology, Prion Research Center, Colorado State University, Fort Collins, Colorado, USA, ^3^ Laboratory of Prion Neurobiology, Department of Neuroscience, Istituto di Ricerche Farmacologiche Mario Negri IRCCS, Milan, Italy, ^4^ Boston University, Department of Chemistry, Boston, Massachusetts, USA), gave the Richard A. Bessen Lecture. They displayed their work on anti-prion treatment using experimental drugs. Prion diseases are fatal neurodegenerative diseases of humans and other mammals with no current treatment options. In this study, they characterized a novel anti-prion compound, elacridar (GW120918), which has sub-micromolar activity in assays of prion infection, propagation, and toxicity. Elacridar acts at an early step in the prion infection process, enhancing degradation of newly formed PrP^Sc^. The lysosome is the likely site of elacridar’s anti-prion effects, based on transcriptomic analysis and the use of functional lysosomal probes. Elacridar alters gene expression networks controlling lysosomal sterol and lipid metabolism, but, unlike other lysosomotropic drugs, it prominently upregulates genes that control lysosomal pH. Surprisingly, these effects occur independently of TFEB nuclear translocation, suggesting novel regulatory mechanisms. The anti-prion effects of elacridar extend to α-synuclein and tau prions, highlighting lysosomal enhancement as a general strategy for treatment of protein-misfolding neurodegenerative diseases. This work was supported by the National Institutes of Health grant number 5R01NS065244, awarded to DAH. RCCM was supported by grants from the Department of Defense (W81XWH-21-1-0141) and the Creutzfeldt−Jakob Disease Foundation. All animal studies were performed following guidelines and protocols approved by the Institutional Animal Care and Use Committee of Boston University.

Diana C, Lowe ^1,2^, in conjunction with Julianna Sun ^1,2^, Sehun Kim ^2^, Jifeng Bian ^2,3^, Jenna Crowell ^2^, Emma Raisley ^2^, Madeline Judson ^2^, Maria Nöremark ^4^, Dolores Gavier-Widen ^4,5^ Sirkka-Liisa Kopenfelt ^6^, Sylvie Benestad ^7^, and Glenn Telling ^1,2^ presented their research on Chronic wasting disease (CWD) (^1^ Cell and Molecular Biology Graduate Program, Colorado State University; ^2^ Prion Research Center, Department of Microbiology, Immunology and Pathology, Colorado State University, ^3^ Virus and Prion Research Unit, National Animal Disease Center, Agricultural Research Service, United States Department of Agriculture, Ames, IA, ^4^ Department of Disease Control and Epidemiology, National Veterinary Institute, Uppsala, Sweden; ^5^ Department of Biomedical Sciences and Veterinary Public Health, Swedish University of Agricultural Sciences, Uppsala, Sweden; ^6^ Finnish Food Authority, Animal Health Diagnostic Unit, 00790, Helsinki, Finland, ^7^ Norwegian Veterinary Institute, Ås, Norway). CWD is a highly infectious prion disease of deer, elk, moose, and other cervids that poses an increasingly unpredictable threat to human health. While the CWD epidemic has existed in North America for decades, CWD recently emerged in Norway, Sweden, and Finland. Their previous studies showed that the strain properties of CWD prions from Norwegian and Finnish cervids were distinct from those of North American CWD. Notably, their studies of European moose CWD suggest a unique strain diversity, reflected in the remarkable heterogeneity of their properties. Recently, they have expanded on these findings with their studies of Swedish moose CWD. She analyzed the transmission properties of the four moose CWD isolates that were reported in Swedish cases in our gene-targeted (Gt) mouse models, which express cervid PrP at physiological levels. She analyzed the kinetics of disease, PrP^Sc^ accumulation in the brain, neuropathology, prion conformational stability, and assessed in vitro amplification properties by RT-QuIC and protein misfolding cyclic amplification (PMCA). Similarly to isolates from moose in Norway and Finland, her team found that Swedish moose CWD isolates have diverse transmission kinetics, including one disease isolate displaying a remarkably rapid onset of disease in only 90 days. Conformational stability, histological features, and amplification properties differed among the Swedish isolates and were distinct not only from North American CWD but also from Norwegian and Finnish moose CWD. They conclude that the strain properties of CWD prions in Northern European moose are highly diverse, and their capacity for adaptation is unpredictable, supporting the hypothesis of a sporadic origin. This research was funded by the following NIH grants: R35NS132226, R01NS121682, and P01AI077774; the qCMB T32 NIH-NIGMS Fellowship GM132057 and the IDRRTP Training Fellowship Award funded by CVMBS-OVPR at Colorado State University. All animal studies were performed following guidelines and protocols approved by the Institutional Animal Care and Use Committee of Colorado State University.

Benjamin S. Steadman, together with Ronald A. Shikiya, and Jason C. Bartz (Department of Medical Microbiology and Immunology, School of Medicine, Creighton University, Omaha, NE, United States of America, ^2^ Department of Microbiology, Immunology, and Pathology, Prion Research Center, Colorado State University, Fort Collins, CO, United States of America), discussed the results of their research on minor prion strains and their relation to hyperexcitability and weight gain. Prions are self-propagating conformations of the prion protein, PrP^Sc^, that result in infectious neurodegenerative diseases. Prion strains differ in heritable protein conformations of PrP^Sc^ that produce unique clinical phenotypes. Prions exist as dynamic quasispecies consisting of a dominant strain and minor prion strains. Recently, pre-existing minor prion strains have been directly observed by biochemically reducing PrP^Sc^ from hamster-adapted drowsy transmissible mink encephalopathy (DY TME) and amplifying the remaining PrP^Sc^ through protein misfolding cyclic amplification (PMCA). Minor prion strains differed from their parental strain, DY TME, in incubation periods, clinical signs, conformational stability, proteinase resistance, cell conversion efficiency, and intraspecies and interspecies PMCA conversion efficiency. As heat can inactivate prions in a strain-specific manner, their objective was to identify heat conditions that enable minor prion strains to emerge. To test this, they exposed DY TME to temperatures ranging from 85 to 105 °C for 24 h and then probed for pre-existing minor prion strains. They found that heat exposure of DY TME resulted in emerging thermostable minor prion strains with shorter incubation periods and different clinical signs. Most transmissible prion strains were hyperexcitable; however, they also identified a pre-existing minor prion strain with progressive weight gain, referred to as heavy (HVY TME). Minor strain PrP^Sc^ also differed from their parental strain, DY TME, in antibody reactivity, thermostability, intraspecies and interspecies PMCA conversion efficiency, and lymphotropism. Collectively, this provides further evidence to support that multiple pre-existing minor prion strains with several strain phenotypes can emerge, highlighting the importance of prion surveillance in preventing further interspecies transmission of fatal prion diseases. The emergence of thermostable minor prion strains is particularly relevant when considering the impact of wildfires upon environmentally transmissible prion diseases, such as chronic wasting disease in cervids. All animal studies were performed following guidelines and protocols approved by the Institutional Animal Care and Use Committee of Creighton University.

### 2.4. Investigating Arboviruses and Their Vectors

Matthew J. Abbott and Alyssa B. Evans, from the Department of Microbiology and Cell Biology at Montana State University, discussed their evaluation of orthobunyavirus genome segments and their involvement in neurovirulence through reassortment. The California serogroup (CSG) of orthobunyaviruses is a group of genetically similar, tri-segmented RNA viruses, seven of which are encephalitic in humans. Encephalitic CSG viruses, including La Crosse virus (LACV) and Inkoo virus (INKV), display disparate neuropathogenic phenotypes despite their high degree of genetic similarity. LACV is the leading cause of pediatric arboviral encephalitis in the USA, and many children who recover from infection experience lifelong neurological sequelae, likely in part due to permanent neuronal death. Conversely, INKV has only caused several confirmed neuroinvasive disease cases, mainly in Scandinavian countries. Murine models of infection recapitulate these phenotypic differences, where LACV has high neurovirulence, and INKV has low neurovirulence. The viral factors that mediate these divergent phenotypes are largely unknown. By leveraging the tri-segmented nature of CSG viruses, they created reassortant viruses between the L, M, and S segments of LACV and INKV to identify the genome segments driving neurovirulence. They characterized the replication kinetics and cytotoxicity of reassortant viruses in human neuronal cells in vitro and evaluated neurological disease and viral replication in vivo in mice. The in vivo results suggest the LACV M segment, encoding two envelope glycoproteins and a nonstructural protein, and the S segment, encoding a nucleocapsid protein and a nonstructural protein, are both key mediators of neurovirulence, but neither segment alone drives neurovirulence. Replication kinetics assays showed some differences in growth kinetics; however, all reassortant viruses replicated to similar end titers. Cytotoxicity assays closely matched in vivo results and suggested the LACV M segment mediates LACV’s ability to efficiently kill neurons. Results suggest that the S segment enhances virulence in vivo. Together, their results indicate viral factors encoded on both the LACV M and S segments are required for high levels of orthobunyavirus neurovirulence. All animal studies were performed following guidelines and protocols approved by the Institutional Animal Care and Use Committee of Montana State University.

Lea Blank, partnering with Christin Lorenz and Imke Steffen (Institute of Biochemistry, University of Veterinary Medicine, Hannover, Germany, ^2^ Research Center for Emerging Infections and Zoonoses, University of Veterinary Medicine, Hannover, Germany, ^3^ Department of Microbiology, Immunology and Pathology, College of Veterinary Medicine and Biomedical Sciences, Colorado State University, Fort Collins, Colorado, USA) presented the results of their research on species-dependent roles of Hsp70 chaperones in orthoflavivirus glycoprotein secretion and virion assembly. Orthoflaviviruses, including tick-borne encephalitis virus (TBEV) and West Nile virus (WNV), are arthropod-borne RNA viruses that can cause severe neurological disease and represent significant public health threats, yet no specific antiviral treatments are available. Two key glycoproteins, the envelope protein (E) and non-structural protein 1 (NS1), are essential components of the orthoflavivirus life cycle. E mediates viral entry by binding to host cells and promoting membrane fusion, while NS1 exists in multiple oligomeric forms, contributing to replication and immune evasion. Both proteins exploit the host endoplasmic reticulum (ER), where heat shock protein 70 (Hsp70) chaperones, including the ER-resident chaperone BiP, regulate protein folding and the unfolded protein response (UPR). They investigated the interactions between orthoflavivirus glycoproteins and Hsp70 chaperones, as well as their functional roles in viral secretion and infectivity. Using co-immunoprecipitation assays, they demonstrated that both E and NS1 proteins interact with Hsp70 chaperones in a species-specific manner. Targeting the nucleotide-binding domain of Hsp70 with a small-molecule inhibitor strongly reduced viral infectivity across multiple orthoflaviviruses, including TBEV and WNV, without significantly altering NS1 secretion. In contrast, inhibition of the Hsp70 substrate-binding domain selectively impaired NS1 secretion in tick-borne orthoflaviviruses, such as TBEV and Langat virus (LGTV), but had little effect on virion release or infectivity. siRNA-mediated knockdown of BiP led to reduced E and NS1 protein expression across several species, while having only minor effects on viral infectivity. These results demonstrate species-dependent differences in how orthoflaviviruses exploit Hsp70 chaperones, revealing critical mechanistic roles in glycoprotein maturation, secretion, and viral infectivity. Such insights not only deepen our understanding of virus–host interactions but also identify Hsp70 as a promising target for antiviral intervention. This research was funded by the Deutsche Forschungsgemeinschaft (DFG; German Research Foundation)—398066876/GRK 2485/2 and the Federal Ministry of Education and Research (BMBF) under project number 01KI1719 as part of the Research Network Zoonotic Infectious Diseases. No animals or human studies were performed.

Mollie Burton, Madison Stoltz, Tyler Sherman, and Christie Mayo (Dept. of Microbiology, Immunology, and Pathology, Colorado State University) discussed the results of their study on cross-sectional prevalence of Orbiviruses in Colorado and surrounding areas in 2022–2023. Bluetongue virus (BTV) and epizootic hemorrhagic disease virus (EHDV) are pathogens vectored by *Culicoides* spp. midges that infect wild and domestic ruminants worldwide. Although endemic in the United States, current and ongoing outbreaks of previously unreported serotypes of both BTV and EHDV in Europe have resulted in significant economic losses and emphasize the need for active surveillance in North America and abroad. This work aims to provide current information regarding the prevalence of these viruses in Colorado and surrounding states. Serum and whole blood were collected from sheep and cattle sites or meat processing facilities during a period of two weeks in October 2022 (n = 17) and 2023 (n = 23). They evaluated these samples using a duplex BTV/EHDV qRT-PCR, BTV cELISA, and EHDV cELISA. Detection of BTV RNA was greater in both cattle and sheep in 2022 (41%, 25% [95% CI: 32–51%, 18–33%]) as compared to 2023 (17%, 4% [95% CI: 13–22%, 1–9%]). Detection of EHDV RNA was greater in cattle than sheep in 2022 (13%, 1% [95% CI: 8–21%, 0–4%]) but negligibly differed between species in 2023 (8%, 7% [95% CI: 5–11%, 2–12%]). The seroprevalence of BTV in sheep flocks increased slightly from 2022 to 2023 (42%, 46% [95% CI: 34–50%, 36–55%]) but decreased in cattle herds between years (63%, 54% [95% CI: 54–72%, 48–59%]). In contrast, seroprevalence of EHDV in sheep flocks increased slightly between years (20%, 25% [95% CI: 14–27%, 17–34%] and decreased in cattle herds between years (60%, 56% [95% CI: 50–68%, 50–62%]. These findings show the continued circulation of BTV and EHDV in domestic livestock populations in Colorado and surrounding areas and highlight the contribution of these ruminants in the maintenance of these viruses. This work will provide empirical data for predictive modeling efforts as the team works towards understanding the ecology of *Orbiviruses* at the wildlife–livestock interface. All animal studies were performed following guidelines and protocols approved by the Institutional Animal Care and Use Committee of Colorado State University.

Corey Campbell, researching in partnership with Hunter A. Ogg, Zoey M. Mikol, David C. King, Chad E. Mire, Zeyad Arhouma, Erin Osborne Nishimura, and Rebekah C. Kading (Dept. of Microbiology, Immunology, and Pathology, Colorado State University) presented the results of their study on how Rift Valley Fever Virus alters the chromatin landscape in Aedes aegypti. When arboviruses infect midgut epithelia of vector mosquitoes, viral components interact with host proteins to hijack cells and initiate replication. Alterations of the mosquito genomic regulatory landscape during arbovirus infection have been largely unexplored. This work defines transcriptional regulatory changes using Rift Valley fever virus (RVFV MP12, *Phlebovirus riftense*, family *Phleboviridae*) in *Aedes aegypti*. Midguts were removed at 1, 3, and 7 days post-exposure (dpe) for RNA-Seq and chromatin immunoprecipitation sequencing via Cleavage Under Targets and Release Using Nuclease (CUT and RUN), using acetylated histone 3 lysine 27 (H3K27ac) and triple methylated H3K9 (H3K9me3) antibodies. The goal was to interrogate chromatin regions and enhancers associated with areas of active gene expression by identifying differentially abundant histone modifications following either infectious or non-infectious bloodmeals. Strikingly, though global patterns showed increased H3K27ac marks upon blood feeding and following RVFV MP12 exposure, significantly differentially bound peaks (DiffBind) showed progressive depletion as infection progressed. Geneset enrichment analysis (GSEA) revealed that general immune response transcripts were enriched at 1 and 3 dpe, whereas hedgehog/GLI signaling pathway transcripts were depleted at 7 dpe. Moreover, at 7 dpe, 7/102 differentially expressed genes (DEGs), coding for a signaling protein and several genes expected to facilitate protein–protein interactions on intracellular membranes (*p* < 0.10, DESeq2), were proximal to differentially acetylated sites (<=2000 nts) in a pattern expected to favor viral propagation. To identify altered genomic regulatory regions upon blood feeding alone, CUT&RUN/RNA-Seq of midguts from blood-fed versus sugar-fed mosquito midguts revealed global changes to H3K27ac and H3K9me3 marks during and following the period of bloodmeal digestion. H3K27ac marks were proximal to one quarter of all DEGs at 1 day post-bloodmeal, consistent with an important role of H3K27ac in the regulation of genes that confer metabolic changes. In contrast, it took several days for H3K27ac marks to be associated with DEGs in RVFV-exposed midguts. No animal or human studies were performed.

Samantha J. Courtney, together with Emily N. Gallichotte, Emma Nilsson, Chasity E. Trammell, Kate X. Kimball, Anna C. Fagre, Allison Vilander, Anna K. Överby, and Gregory D. Ebel, presented their research on identifying Powassan virus (POWV) amino acids that contribute to disease phenotypes in mice. Powassan virus is an emerging tick-borne flavivirus that causes severe neurologic disease in humans. POWV has considerable genetic and phenotypic diversity, including highly variable pathogenesis in mice. This study investigated the phenotypic variability within lineage II strains in C57BL/6 mice. Relative to other strains, two New York isolates, NY.19.12 and NY.19.32, caused earlier clinical signs and detection of viral RNA (vRNA) in the spleen and brain compared to mice infected with a lineage II infectious clone (WI.97.ic). Notably, NY.19.12 and NY.19.32 share three amino acid mutations in envelope, NS1, and NS5 compared to other lineage II strains, which were engineered into an infectious clone (NY.19.12.ic). They characterized infection at six days post-infection and disease endpoint for WI.97.ic, NY.19.12, and NY.19.12.ic strains. At early time points, clinical scores, vRNA detection in the cerebellum, and viral distribution in the brain were similar between NY.19.12 and NY.19.12.ic, indicating these mutations may play a role in early neuroinvasion and overall disease progression. However, NY.19.12 vRNA was detected in the spleen at significantly higher rates compared to both WI.97.ic and NY.19.12.ic, indicating factors other than these mutations are responsible for increased spleen infection. Importantly, this study highlights the complexity of POWV pathogenesis and suggests that POWV lineage II strains have varying disease phenotypes likely driven by multiple genetic differences. This research was funded by the National Institute of Allergy and Infectious Diseases (R01AI137424). SC was supported by funding from the National Institute of Allergy and Infectious Diseases (T32AI162691). All animal studies were performed following guidelines and protocols approved by the Institutional Animal Care and Use Committee of Colorado State University.

Jack Dorman, along with William Bakhache and Patrick T. Dolan from the Quantitative Virology and Evolution Unit, Laboratory of Viral Diseases, NIH-NIAID Division of Intramural Research, Bethesda, MD, presented his work “Through Taxa and Time”. The structural proteins of flaviviruses are subjected to complex selective pressures, given that they balance fitness in vertebrate and arthropod hosts. Of these pathogens, West Nile virus (WNV) is the most common in the United States. Recent results have demonstrated adaptation to different hosts as the virus has spread globally, but findings are largely dependent on public health surveillance, which is sporadic and covers only recent history. To provide more systematic understanding of how different host environments shape diversity in flaviviruses, they performed deep mutational scanning (DMS) on a key WNV protein, the Envelope (E) protein. They screened our full DMS library in bird (the predominant vertebrate host), human, and mosquito cells. They demonstrated how constrained E is and the sites of functional trade-off between the three hosts. They further compared constraints to natural populations of WNV and other flaviviruses to show the extent to which DMS results account for natural diversity. Their analysis shows how effectively constraint is captured across many mosquito-borne taxa, but it begins to break down as we include tick-borne viruses. To understand how constraints were navigated historically, they performed ancestral sequence reconstruction (ASR) on WNV E and generated 38 intermediate amino acid substitutions going from the common ancestor to fourteen contemporary isolates. Screening this library on the three host cells of our DMS screen demonstrates that host tropism was dynamic across recent evolutionary history but is more stable with more conserved substitutions. The combined power of DMS and ASR maps fitness across phylogenetic scales, increasing our understanding of how such extreme fitness landscapes are navigated. These insights may enhance our understanding of the process of arbovirus emergence. This research was supported by the Intramural Research Program of the National Institutes of Health (NIH) under project 1ZIAAI001360. The contributions of the NIH authors were made as part of their official duties as NIH federal employees, follow agency policy requirements, and are considered Works of the United States Government. However, the findings and conclusions presented in this paper are those of the authors and do not necessarily reflect the views of the NIH or the U.S. Department of Health and Human Services. No animal or human studies were performed.

Uriel Enrique Aquino Ruiz ^1,2,3^, alongside Katharina Rahmel ^3,4^, Antonio A. R. Camarão ^1,2^, Andreas Pavlou ^4^, Ulrich Kalinke ^4^, Imke Steffen ^1,2,3,5^ (^1^ Institute of Biochemistry, University of Veterinary Medicine Hannover, Hannover, Germany; ^2^ Research Center for Emerging Infections and Zoonoses, University of Veterinary Medicine Hannover, Hannover, Germany; ^3^ Hannover Biomedical Research School, Hannover Medical School, Hannover, Germany; ^4^ Institute for Experimental Infection Research, Centre for Experimental and Clinical Infection Research, TWINCORE, Hannover, Germany; ^5^ Center for Vector-Borne Infectious Diseases, Department of Microbiology, Immunology and Pathology, Colorado State University, Fort Collins, CO, USA), discussed their research on how secreted high molecular weight glycoprotein of orthoflaviviruses modulates innate immune responses in dendritic cells. Orthoflaviviruses are arthropod-borne viruses transmitted by mosquitoes or ticks, responsible for diseases ranging from asymptomatic to hemorrhagic fever and encephalitis with long-term neurological and cognitive repercussions. Orthoflaviviruses are characterized by a single-stranded positive-sense RNA genome of approximately 11 kilobases that encodes a single polyprotein. This polyprotein is post-translationally cleaved into three structural and seven non-structural proteins. Among these proteins, the non-structural protein 1 (NS1) is a key factor for viral replication, immune evasion, and pathogenesis. NS1 is secreted from infected cells as a soluble high-density lipoprotein and plays a crucial role in viral dissemination while also modulating the host immune response. Recent research from their group has reported the immunomodulatory effects of sNS1 proteins from orthoflaviviruses on primary dendritic cells in a sterile inflammation model. However, the precise mechanism underlying this modulatory function by sNS1 during orthoflavivirus infection remains unknown. In this study, they investigated the impact of sNS1 from tick-borne encephalitis virus (TBEV) and West Nile virus (WNV) on monocyte-derived dendritic cells moDCs) upon the homologous infection. In their experiments, human moDCs from several healthy donors were pretreated with TBEV-sNS1 or WNV-sNS1 for 16 h before being infected with TBEV (strain Neudoerfl), WNV (strain NY99), or stimulated with polyinosinic-polycytidylic acid (poly(I:C)). Cell lysates were collected for Western blot analysis to detect the expression of the RIG-I-like receptor (RLR) melanoma differentiation-associated protein 5 (MDA-5). Remarkably, sNS1 pretreatment significantly suppressed MDA-5 and RIG-I activation in response to both infection and poly(I:C) stimulation, orthoflavivirus infection and poly(I:C) stimulation in comparison with infection or poly(I:C) without sNS1 exposure. Their findings demonstrate the potent immunoregulatory role of sNS1 proteins in disrupting innate immune signaling in dendritic cells. This study provides new insights into how highly pathogenic orthoflaviviruses manipulate dendritic cell-mediated innate immune responses, offering potential avenues for therapeutic intervention in orthoflavivirus-induced diseases. No animal or human studies were performed.

Anna Fingalson, along with Miles Stroud and Corey L. Campbell from the Department of Microbiology, Immunology, and Pathology, Colorado State University, shared her research on CUT&RUN investigations of SREBP regulation in *Culex tarsalis* mosquitoes. Blood feeding in mosquitoes triggers a complex transcriptional response that is about 14% of overall gene expression, yet many underlying regulatory mechanisms remain poorly understood. Rift Valley Fever Virus (RVFV), an arbovirus, triggers a series of biochemical changes crucial for the virus’s replication and transmission when it infects the midguts of mosquitoes. Understanding how blood feeding alters mosquito midguts is therefore critical for uncovering the molecular pathways that facilitate pathogen replication and transmission. Sterol regulatory element-binding proteins (SREBPs) are transcription factors that regulate lipogenic and cholesterogenic gene expression. Within the nucleus, SREBPs interact with specific transcriptional cofactors, which can enhance or suppress their transcriptional activity. This work explores changes to SREBP binding patterns on chromatin induced by blood feeding in *Culex tarsalis.* Midgut tissues were collected one day post-bloodmeal and analyzed using Cleavage Under Targets and Release Using Nuclease (CUT&RUN). CUT&RUN utilizes a specific primary antibody and protein A–protein G–micrococcal nuclease to isolate chromatin bound to anti-SREBP antibody. A negative control antibody was used to validate the specificity of protein–DNA interactions. Their goal is to identify changes in genomic regulatory regions that are correlated to active gene expression following blood feeding, thereby contributing to a better understanding of the metabolism of mosquitoes and vector biology. The results are expected to reveal the impact a bloodmeal has on the expression of SREBPs, leading to improved insight into the regulation of lipid homeostasis. All animal studies were performed following guidelines and protocols approved by the Institutional Animal Care and Use Committee of Colorado State University.

Alina Friedrichs, with Imke Steffen from the Institute for Biochemistry, University of Veterinary Medicine Hannover Research Center for Emerging Infections and Zoonoses, University of Veterinary Medicine Hannover, Center for Vector-Borne Diseases, and Dept. of Microbiology, Immunology, and Pathology, Colorado State University, presented her research titled “Induction and Evasion of Type-I Interferon and Downstream Antiviral Effects by Neurotropic, Tick-borne *Orthoflaviviruses*”. Although tick-borne orthoflaviviruses exhibit high structural and genetic similarity, they differ significantly in their pathogenicity and host cell tropism. Tick-borne encephalitis (TBEV) is a neurotropic virus linked to severe cases of meningoencephalitis throughout Europe and Asia. In contrast, the closely related Langat virus (LGTV) has not been associated with any natural human infections. The mechanisms underlying the differences remain elusive. This study compares high- and low-pathogenic tick-borne orthoflaviviruses in their ability to induce, modulate, and evade Type-I interferon (IFN) signaling. Additionally, they aim at identifying novel restriction factors of these viruses. Human SNB-19 astrocytoma cells were infected with LGTV and two strains of TBEV, Neudoerfl and Hypr. Supernatants were analyzed to quantify infectious particles and secreted IFN-β levels. Cell lysates were collected for protein analysis by Western blot, as well as RNA extraction and subsequent RT-qPCR. Both TBEV strains productively infected SNB-19 cells, whereas LGTV exhibited slower replication and only produced significantly lower titers. In the first 24 h of the infection, all viruses induced comparable levels of IFN-β. However, at later time points, IFN-β levels of TBEV-infected cells exceed those of LGTV-infected cells by up to a 10-fold. Surprisingly, downstream signaling—as indicated by phosphorylated STAT1 (pSTAT1)—did not correspond to this pattern. High levels of pSTAT1 could be detected in LGTV-infected cell lysates, low levels in TBEV Hypr-infected cell lysates, and pSTAT1 was undetectable in TBEV Neudoerfl. The expression of antiviral interferon-stimulated genes (ISGs) was consistent with this. Their findings suggest that both TBEV strains antagonize the JAK/STAT pathway to suppress ISG induction and evade the host cell antiviral state. In contrast, LGTV permits IFN signaling, which may restrict its replication. Treatment with a selective JAK inhibitor significantly enhances LGTV titers over the course of 72 h, further supporting our findings. No animal or human studies were performed.

Charlize Geer, alongside Marylee Kapuscinski, Audrey Bankes, Brett Moehn, and Mark Stenglein from the Cell and Molecular Biology Program, Colorado State University, and the Department of Microbiology, Immunology, and Pathology, Colorado State University, presented their work on how Orthobunyavirus L-N protein interactions limit reassortment. Viral reassortment occurs when two different segmented viruses co-infect a cell, and the viral genomes are mixed to produce a novel virus. Reassortment drives the evolution of viruses and is a major source of new and emerging human pathogens. The Orthobunyavirus genus contains viruses with a tri-segmented negative-sense RNA genome that encodes several proteins, including a nucleoprotein (N protein, encoded on the S segment) and polymerase (L protein, encoded on the L segment). During orthobunyavirus reassortment, L and S segments tend to reassort together, indicating that mismatched L and N proteins do not function together. However, the molecular basis for this incompatibility is unknown. To understand the molecular details of L and N protein compatibility, they used a minigenome assay to test the function of La Crosse virus (LACV) L protein with N proteins from 50 different orthobunyaviruses that spanned a broad range of relatedness to LACV N. They found that N proteins sharing ~70% or greater amino acid sequence identity with LACV N were able to function with the LACV L protein, while sequences with lower identity showed no function. Colabfold implementation of Alphafold2 was used to generate predicted N protein structures, which help visualize the findings of our minigenome assay. Understanding this critical part of the virus life cycle could lead to a new target for therapeutics and will inform predictions of reassortment risk between different orthobunyaviruses. No animal or human studies were performed.

Leonardo Giordano, along with Jack Dorman and Patrick T. Dolan from the Quantitative Virology and Evolution Unit, Laboratory of Viral Diseases, NIH-NIAID Division of Intramural Research, Bethesda, MD, presented his research on differences in thermal stability of dengue virus serotypes 1–4. Flaviviruses are a diverse group of RNA viruses responsible for significant global mortality. Among them, dengue virus (DENV) remains a leading public health threat, with four different serotypes (DENV-1 to DENV-4) causing over 390 million infections annually. Temperature is a critical environmental factor influencing viral infectivity and stability, with important implications for outbreak prediction and vaccine development. However, traditional methods to quantify viral infectivity are often labor-intensive and prone to variability, limiting their scalability for comparative studies across viral strains and conditions. To address this, they developed a high-throughput, semi-automated workflow using open-source liquid-handling robots (Opentrons OT-2 and Flex) to standardize the focus forming assay (FFA), a standard method for quantifying infectious flavivirus particles. Their automated pipeline performs the entire FFA process, including the serial dilution of virus stocks, automated plating and infection of cells, and fixation and antibody immunostaining steps. This automated pipeline reduces hands-on time, minimizes user exposure to the virus, and ensures consistent sample handling, significantly improving reproducibility and scalability. Using this automated pipeline, they assessed temperature-dependent decay assays across all four DENV serotypes. Preliminary data show serotype-specific differences in temperature stability, with critical inactivation temperatures ranging from 43 to 47 C, suggesting significant diversity in thermostability phenotypes. These phenotypes are consistent with the phylogenetic relationship of these serotypes, suggesting they derive from ancestral genotypes. Their integrated approach combining automation with a phenotypic assays approach improves the scalability of phenotypic assays and provides important insights into how temperature shapes DENV stability, with potential applications in dengue control and vaccine strategies. This research was supported by the Intramural Research Program of the National Institutes of Health (NIH) under project 1ZIAAI001360. The contributions of the NIH authors were made as part of their official duties as NIH federal employees, are in compliance with agency policy requirements, and are considered Works of the United States Government. However, the findings and conclusions presented in this paper are those of the authors and do not necessarily reflect the views of the NIH or the U.S. Department of Health and Human Services. No animal or human studies were performed.

Michelle Law and Patrick T. Dolan from the Quantitative Virology and Evolution Unit, Laboratory of Viral Diseases, NIH-NIAID Division of Intramural Research, Bethesda, MD, shared their research titled “Unraveling dengue virus NS5 linker in and domain evolution”. Dengue virus (DENV) is an enveloped, positive-sense RNA virus and a member of the orthoflavivirus genus consisting of four serotypes. It is a medically important arbovirus with a significant disease burden globally. Its genome encodes seven nonstructural (NS) proteins, which are involved in viral replication. Nonstructural protein 5 (NS5), is the largest and most conserved flaviviral protein comprising a methyltransferase (MTase) domain and RNA-dependent RNA polymerase (RdRp), connected by a flexible nine-residue linker. This linker plays a crucial but poorly understood role in coordinating interdomain interactions during viral replication. This project aims to dissect how the DENV NS5 linker regulates replication complex function across serotypes using deep mutational scanning (DMS), cell-based assays, and structure–function characterization. To support this work, they demonstrate the development of a cell-free system to streamline mutant virus generation and minimize challenges with flavivirus reverse genetics. They have also generated NS5 hybrid viruses whereby NS5 in the DENV2 16681 strain is replaced with NS5 from other serotypes. These hybrids allow DMS screening to be extended to different serotypes without having to rescue infectious clones of every serotype. They also provide an opportunity to investigate how interprotein interactions between NS5 and the other nonstructural proteins might contribute to serotype chimera incompatibility. The impact of this work will advance our understanding of dengue virus genome synthesis and capping, and more broadly, viral replication and fitness in different hosts. This research was supported by the Intramural Research Program of the National Institutes of Health (NIH) under project 1ZIAAI001360. The contributions of the NIH authors were made as part of their official duties as NIH federal employees, are in compliance with agency policy requirements, and are considered Works of the United States Government. However, the findings and conclusions presented in this paper are those of the authors and do not necessarily reflect the views of the NIH or the U.S. Department of Health and Human Services. No animal or human studies were performed.

Austin J. Mejia ^1^, alongside Nang Phyu Phwe ^2^, Pyae Phyo Aung ^2^, Thura Soe Min Htike ^2^, Tin Htun Aung ^2^, Ohnmar Aung ^3^, Juliette Lewis ^1^, Emma Harris ^1^, Nicole Gardner ^3^, Tierra Smiley Evans ^3^, Rebekah C. Kading ^1^, Christine Kreuder-Johnson ^3^ (^1^ Center for Vector-borne Infectious Diseases, Dept. of Microbiology, Immunology, and Pathology, Colorado State University, Fort Collins, CO, ^2^ Nature Conservation Society, Myanmar, ^3^ One Health Institute, School of Veterinary Medicine, University of California, Davis, CA) presented his research on mosquito surveillance across habitats in Myanmar and Kaeng Khoi virus host plasticity. Myanmar is considered one of the most biodiverse countries in the Asia-Pacific region. Yet, its mosquito population and role in arbovirus transmission are greatly understudied. To address this knowledge gap, they conducted mosquito surveillance in Myanmar across a landscape gradient consisting of forest, edge, and urban habitats. They hypothesize that virus–host plasticity increases at edge habitats where vertebrates and mosquitoes interact. They collected 36,194 mosquitoes from forest, edge, and urban habitats across the wet and dry seasons and will be analyzing species diversity, bloodmeal source, and arbovirus presence. Furthermore, they examined Kaeng Khoi virus (KKV), an understudied orthobunyavirus originally found in Thailand’s wrinkle-lipped free-tailed bats (*Mops plicatus*), as a model to assess viral host plasticity. Using in vitro growth curve experiments, they tested KKV replication in mosquito (Aag2, C6/36), bat (AJK-6, TB1 LU), human (HUH-7), and primate (Vero) cell lines. Cells were infected at an MOI of 0.1, and viral titers were measured over 96 h using plaque assays. They hope to utilize their data to understand Myanmar’s mosquito and arboviral diversity, how mosquitoes interact with different hosts across habitats, how KKV replicates in various species cells, and the role of host plasticity in arbovirus transmission. This work was funded by award number 2109860 from the National Science Foundation to the University of California at Davis, through subaward A22-0516-S001 to Colorado State University.

Elizabeth Mielke, along with Ogg, Hunter, Kading, R.C., Wilson, W.C., Mire, C., Fitzmeyer, L., Hill, Jessica, and Campbell, C.L., presented their work on the identification of somatic cell-type markers in *Culex tarsalis.* Rift Valley Fever Virus (RVFV) causes a mosquito-borne viral disease that affects livestock and humans, primarily in sub-Saharan Africa, occasionally resulting in mass animal die-offs. These outbreaks can lead to devastating economic and food security losses for agricultural communities. Over 40 mosquito species can potentially act as transmission vectors. RVFV is retained in nature partially through vertical transmission, which they hypothesize begins with a disseminated viral infection of the adult female and moves to the developing eggs. Previous reports indicated that approximately 30% of a mosquito’s total transcriptomic response is in the ovaries, leading to the following question: What cell types constitute a mosquito ovary? Thus, they chose *Culex tarsalis,* a major vector for West Nile virus in North America and highly competent for transmission of RVFV, as our subject for single-cell RNA sequencing (scRNA-seq) analysis. The objective of this project is to identify expressed marker genes for ovarian somatic cell types that are potentially involved in vertical transmission. Ovarian single-cell suspensions were prepared from pools of 20 sets of ovaries in duplicate. The Chromium Next GEM Single Cell 3’ Reagent kit was used to create libraries for scRNA-seq of adult ovaries that had already laid one round of eggs. Cell Ranger and Seurat cluster analyses were used to identify 13 significant cell clusters, the top 100 expressed genes per cluster, and putative ovarian cell types for each cluster. Stretch cell marker SGPL1 (sphingosine-1- phosphate lyase), hemocyte cell markers ALDH18A1 (delta-1-pyrroline-5-carboxylate synthase) and APO (apolipophorins), nurse cell marker VGR (Vitellogenin Receptor), and mitotic follicle cell marker CORIN (Corin, serine peptidase) were determined by RT-qPCR differential expression analysis and/or smiFISH. The advances made here for improving the *Culex tarsalis* ovarian transcriptome and defining marker genes enhance the foundational knowledge for vector biologists investigating vertical transmission of RVFV in *Culex tarsalis*. No animal or human studies were performed.

Andrew Muñoz Gamba and Cara Pager presented their research on Zika virus adaptation in mammalian and mosquito cells and how they show differences after ten passages. Zika virus (ZIKV) is a re-emergent flavivirus that was introduced into the Americas in 2015. Zika virus (ZIKV) is a re-emergent flavivirus that was introduced into the Americas in 2015. ZIKV is a mosquito-borne arbovirus transmitted by Aedes aegypti mosquitoes to other species, including humans. Human clinical outcomes range from microcephaly in newborns to Guillain–Barré Syndrome in adults. Unfortunately, no treatments or a vaccine have been approved. In the viral genome, in the NS5 gene, the RNA-dependent RNA polymerase (RdRp) is an error-prone polymerase that introduces mutations during viral replication. As the virus alternates between the mosquito and mammalian hosts, mutations accumulate in the new virions. However, it is still unclear whether these new mutations promote or restrict host adaptation. The goal in this work is to identify genetic determinants of ZIKV during host adaptation. To this end, they performed evolutionary passaging of ZIKV infection in mammalian (African monkey green-Vero) and mosquito (Aedes albopictus-C6/36) cells. The infections were carried in a single host (only mammalian/mosquito cells) or host-switching (alternating between both hosts). Specifically, he passaged ZIKV ten times at a multiplicity of infection of 0.01 plaque-forming units/cell for 72 h post-infection (hpi) in Vero cells and 96 hpi in C6/36 cells. Using plaque assays to phenotypically characterize the virus infections, they found that ZIKV viral titers increased in mammalian cells by one-fold and decreased in mosquito cells by one to two-fold in either single-host or host-switching. These findings show that single and host-switching infections efficiently produce infectious particles. These phenotypic differences suggest the presence of a diverse viral population and likely host adaptation. Next, he will identify the quasispecies landscape associated with host adaptation using next-generation deep sequencing analysis of the complete ZIKV genome. Moreover, they will undertake functional studies to test specific mutations in the context of the viral life cycle, which could uncover potential viral targets for future antivirals or vaccine development. No animal or human studies were performed.

Christina Norsten, together with Alyssa B. Evans, the Department of Microbiology and Cell Biology at Montana State University, presented their work on characterization of gene expression in microglia in response to California Serogroup orthobunyaviruses with differing neuropathogenesis. The California Serogroup (CSG) of orthobunyaviruses is a group of 18 closely related mosquito-borne viruses, seven of which can cause neurological disease in humans. La Crosse (LACV) and Inkoo (INKV) CSG viruses can be encephalitic in humans. LACV is the leading cause of pediatric arboviral encephalitis in the USA, whereas INKV causes little human disease. Similarly, in mice, previous studies have shown that LACV causes neurological disease in all intranasally inoculated mice, whereas INKV causes neurological disease in a few mice. Immunohistochemistry analysis of the brains of inoculated mice showed a greater microglia response to INKV than LACV; neither virus infected microglia. Microglia are the primary resident immune cells of the brain and can play both inflammatory and anti-inflammatory roles, and can be either protective or pathogenic during viral infection. The role of microglia during CSG virus infection is not known. To investigate the role of microglia in CSG virus infection, this research group compared infection and gene expression responses in the human microglia cell line C20 to LACV and INKV. They reported that the microglia were highly susceptible to infection with LACV but less susceptible to infection with INKV. The INKV-infected microglia expressed higher levels of microglia marker Tmem119 and cell proliferation markers Ube2c, Cks1b, and Mki67 compared to LACV-infected microglia. Because microglia are not actively infected in vivo, the research group then exposed microglia to UV-inactivated LACV and INKV. They observed differences in activation and suppression markers and cell proliferation gene expression between LACV and INKV-exposed cells. Notably, there was an increase in activation markers Iba1 and CD68 in response to INKV but not LACV. Together, their results indicated that human microglia preferentially proliferate and activate in response to INKV, but not LACV. This suggested that there is a protective microglia response to INKV infection that is absent during LACV infection. All animal studies were performed following guidelines and protocols approved by the Institutional Animal Care and Use Committee of Montana State University.

Kylee Pham presented her research on the role of ceramide synthase 2 in the dengue virus life cycle. Suad Elmegerhi, Paul S. Soma, and Rushika Perera, who were at the Center for Vector-borne Infectious Diseases, also contributed to this work. Dengue viruses (DENVs) are flaviviruses. They cause an estimated 390 million infections annually, affecting over 100 countries in tropical and subtropical regions. Kylee and her group have shown that throughout their life cycle, DENVs exploit the host sphingolipid metabolism to promote viral replication. Of the sphingolipids in the cell, ceramide forms the primary metabolic hub. Ceramide is generated in the endoplasmic reticulum by ceramide synthases (CerS) that add a fatty acyl group to sphinganine, ultimately resulting in ceramide. Of the six CerS enzymes, CerS2 most directly affects viral replication complexes due to its role in producing ceramides with very long-chain fatty acids that are essential in cellular membranes. siRNA-mediated loss of function of CerS2 in human liver cells (Huh7s) showed an increase in infectious DENV2 titer. To further investigate the role of ceramides and CerS2 during infection, the research group inhibited ceramide synthesis using a chemical inhibitor, Fumonisin B1 (FB1), in Huh7s and showed a similar phenotype to the siRNA studies. Conversely, they also treated DENV2-infected Huh7s with varying amounts of ceramide and showed that ceramide inhibited DENV2 replication in a dose-dependent manner. Additionally, mRNA and protein expression of CerS2 were examined using qRT-PCR and Western blots, respectively, throughout the course of infection. Their findings show that CerS2 activity is critical for regulating DENV replication, highlighting CerS2 as a potential target for antiviral strategies against dengue. No animals or human studies were performed. This research was funded by the Anschutz Family Foundation and CSU’s Department of Microbiology, Immunology, and Pathology Undergraduate Research Fellowship.

Oshani Ratnayake, together with Irma Sanchez Vargas, Samantha M. Pinto, Paul S. Soma, Barbara Graham, Nunya Chotiwan (Chakri Naruebodindra Medical Institute, Faculty of Medicine Ramathibodi Hospital, Mahidol University, Thailand), Elizabeth McGraw (Department of Biology, Center for Infectious Disease Dynamics, The Huck Institutes of the Life Sciences, Pennsylvania State University, State College, PA), and Rushika Perera (Center for Vector-borne Infectious Diseases, Dept. of Microbiology, Immunology and Pathology, Colorado State University, Fort Collins, CO), presented their work on the role of triglycerides during dengue virus infection in *Aedes aegypti* mosquitoes. Arboviruses such as dengue (DENV), Zika (ZIKV), and chikungunya (CHIKV) are transmitted by *Aedes aegypti* mosquitoes. Using systems biology, this group has shown that these three arboviruses significantly alter the metabolome of these mosquitoes during infection. A key observation in their data was that all three viruses induced unique metabolic environments in the mosquito, indicating diverse virus-vector interactions. Among the altered metabolites, triglycerides (TGs) changed significantly during infection with all three viruses; however, their temporal increase was virus-specific. TGs play a significant role in mosquito biology, including energy metabolism, storage, and vitellogenesis. Oshani and her colleagues hypothesize that triglyceride alteration is required for viral replication or for mounting an immune response. TGs are synthesized by the catalytic activity of diacylglycerol acyltransferase-1 (DGAT-1) enzyme in mosquitoes. Inhibitor-mediated suppression of DGAT-1 in C6/36 (*Ae. albopictus*) cells resulted in a reduction in released virus titers in a concentration-dependent manner. Genome replication was also reduced in the treated cells. Suppression of TGs using dsRNA or an inhibitor against DGAT-1 did not have an impact on midgut infection, midgut escape, dissemination, or transmission of the virus in mosquitoes. However, suppression of TG synthesis impaired egg production in mosquitoes during later reproductive cycles, depicting the critical need for TGs in the biology of the vector. The researchers observed that suppression of TG synthesis using inhibitors or dsRNA was not sufficient to reduce TG levels in the studied systems; therefore, they are currently developing a DGAT-1 knock-out mosquito model using CRISPR/Cas. They anticipate these studies will reveal new roles for this important class of lipids in mosquito biology and in virus–vector interactions. This study was funded by the NIH-NIAID grant R01AI151166. No animal or human studies were performed.

Paul S. Soma discussed his work on the metabolic impact of persistent viral infections in mosquitoes. Irma Sanchez-Vargas, Oshani C. Ratnayake, Samantha M. Pinto, and Rushika Perera at the Center for Vector-borne Infectious Diseases, Department of Microbiology, Immunology, and Pathology, and Center for Metabolism of Infectious Diseases, Colorado State University, Fort Collins, CO, were the other contributors to the study. Mosquitoes are vectors of various viruses and have a significant impact on public health. Virus infection of the mosquito is persistent, meaning once infected, mosquitoes remain infectious for their lifespan. Viruses usurp mosquito metabolic processes to promote the viral lifecycle, e.g., energy/signaling requirements, and synthesis of structural components of virus particles. Understanding the long-term impact of chronic viral infection on mosquito metabolomes can benefit antiviral research and vector control. The group measured the metabolome, lipidome, and virus titer of individual mosquitoes for up to 21 days after infection with two flaviviruses: dengue virus (DENV) and Zika virus (ZIKV), and chikungunya virus (CHIKV), an alphavirus. The metabolic effects of each virus with disparate replication kinetics were related to normal mosquito aging of uninfected mosquitoes. During the experiment, *Ae. aegypti* (Poza Rica) were provided sugar (non-fed), non-infected blood (mock), or a virus-spiked blood meal. Mosquitoes were collected at 0, 5, 7, 14, and 21 days post-infection (DPI). Whole mosquitoes were screened for infection, and virus genome copies were measured by RT-qPCR for infected mosquitoes. Bligh-Dyer extraction on ten infected mosquitoes per condition was followed by untargeted metabolomics/lipidomics via RPLC-MS/MS. Paul discussed that, when compared to sugar-fed mosquitoes, blood-fed (mock or infectious) mosquitoes at 0 DPI had a significantly altered metabolome due to an undigested blood meal. Uninfected (mock) mosquitoes had relatively high phosphocholine levels at 0 DPI, which decreased with time, but relatively higher levels were maintained in infected mosquitoes through 21 DPI, suggesting that phosphocholines decrease with mosquito age, but viruses may require the mosquito to maintain higher metabolic activity. Lyosphosphocholine trends generally were the inverse of phosphocholine trends. Disparate glycerolipid levels were observed between viruses, where DENV had markedly low triglyceride levels at 0 DPI, ZIKV had decreased triglycerides at 21 DPI, and CHIKV had elevated triglycerides across multiple DPI. Diglyceride trends generally followed those of triglycerides. No animal or human studies were conducted.

Haley L. Stuckmeyer together with Cadence B. Allen ^1^, David L. Donermeyer ^1^, David A. Price ^3^, Farheen Fatma ^1^, Rachael E. Rush ^2^, Reena Thakur ^1^, Amy L. Hartman ^2^, Daisy W. Leung ^3^, Gaya K. Amarasinghe ^1^ (^1^ Department of Pathology and Immunology, Washington University School of Medicine in St. Louis, St. Louis, MO, USA. ^2^ Center for Vaccine Research, School of Medicine, University of Pittsburgh, Pittsburgh, PA, USA; Department of Infectious Diseases and Microbiology, School of Public Health, University of Pittsburgh, Pittsburgh, PA, USA. ^3^ Department of Medicine, Washington University School of Medicine, St. Louis, MO, United States) presented her and her group’s work on the role of low-density lipoprotein (LDL) receptors in bunyaviral infection. Haley explained how many LDL receptors have recently been identified as critical host factors for a wide range of virus families, including alphaviruses, flaviviruses, enteroviruses, and betacoronaviruses. These LDL receptors are broadly expressed at the membrane across tissues and contribute to critical physiological functions—from lipid transport and immune regulation to maintaining the permeability of the blood–brain barrier. The role of these proteins as highly promiscuous endocytic receptors suggests a potential shared mechanism of viral infection across diverse viral families. However, each LDL receptor has key structural and functional features that are likely targeted by different viruses. Previous work in her group characterized the receptor low-density lipoprotein receptor-related protein 1 (Lrp1) as a key entry factor for the bunyavirus, Rift Valley Fever Virus (RVFV), in mammals. Additionally, their recent observations suggest that other LDL family proteins may also play a role in RVFV infection and that these receptors could be important for viral entry into invertebrate hosts. In this study, Haley discussed the molecular basis for potential redundancy and mechanisms of viral specificity of these host factors that promote bunyavirus infection. This work has the potential to identify previously unrecognized common therapeutic targets for a wide range of viral infections. No animal or human studies were performed.

Ryan Thompson^1^ together with Rebekah C. Gullberg ^1^, Nunya Chotiwan ^1^, M. Nurul Islam ^1^, Laura A. St Clair ^1^, Elena Lian ^1^, Thomas J. Edwards ^2^, Sudip Khadka ^3^, Christopher Teng ^2^, Barbara Graham ^1^, Kirsten Krieger ^1^, Amber Hopf-Jannasch ^4^, Douglas J. LaCount ^3^, John T. Belisle ^1^, Richard J. Kuhn ^2^, and Rushika Perera ^1^ (^1^ Department of Microbiology, Immunology and Pathology, Colorado State University, Fort Collins, CO, USA, ^2^ Department of Biological Sciences, Purdue University, West Lafayette, IN 47906, ^3^ Department of Medicinal Chemistry and Molecular Pharmacology, Purdue University, West Lafayette, IN 47906, ^4^ Metabolite Profiling Facility (MPF), Bindley Bioscience Center, Purdue University, W. Lafayette, Indiana, United States of America) explained how metabolic shifts can be exploited to drive dengue virus elimination. The advantage derived from microengineering the host cell lipidome and metabolome in order to facilitate replication is a powerful tool that many viruses, including dengue viruses (DENVs), have at their disposal. Currently, there are no antiviral treatments for infection with these viruses. It is critical to identify how viral infection changes the host microenvironment if we are to design any drugs to counteract infection. Using liquid chromatography mass spectrometry (LC-MS), Ryan and his colleagues determined that de novo fatty acid biosynthesis was upregulated during DENV, serotype 2 infection. They also determined that a key enzyme in the pathway, Fatty Acid Synthase (FAS), was recruited to sites of DENV2 viral replication and activated by NS3, a viral protein, to support viral replication. However, inhibiting FAS with Orlistat in DENV2-infected cells did not cause a dramatic decrease in viral titer. LC-MS analysis of Orlistat-treated cells revealed that the purine salvage pathway was elevated in response to Orlistat treatment, suggesting that this may be a compensatory response to rescue viral replication when FAS is targeted. Combined inhibition of the de novo fatty acid synthesis pathway and purine salvage pathway with Orlistat and 6-Thioguanine led to a highly pronounced decrease in DENV2 titer. Their findings demonstrate that lipidomics and metabolomics can identify hotspot metabolic pathways that are altered during DENV2 infection and subsequent inhibitor treatments, leading to an increased efficacy in identifying effective combination therapeutics. No animal or human studies were conducted.

Madeline Yunker^1^ together with Imke Steffen^1,2^ (^1^ Center for Vector-Borne Infectious Diseases, Department of Microbiology, Immunology, and Pathology, Colorado State University, Fort Collins, CO, USA, ^2^ Research Center for Emerging Infections and Zoonoses and Institute of Biochemistry, University of Veterinary Medicine, Hannover, Germany) presented their work on oligomeric diversity of secreted NS1 in tick-borne orthoflaviviruses and implications for transmission. Tick-borne orthoflaviviruses (TBOV), such as Powassan virus and tick-borne encephalitis virus, are emerging human pathogens that can cause severe neuroinvasive disease. Despite their impact, the molecular mechanisms underlying their transmission and pathogenesis are poorly understood. A central player in orthoflavivirus biology is the non-structural protein 1 (NS1), which is essential for the formation of replication complexes and secreted from infected cells in various oligomeric forms that are known to modulate host immune responses. While secreted NS1 (sNS1) is often described as a hexamer, recent work with dengue virus revealed that hepatocytes release NS1 primarily as dimers bound to high-density lipoproteins. This suggests that the oligomeric form of sNS1 is cell-type dependent, with important functional implications. Preliminary data presented by Madeline supported this hypothesis, showing variation in TBFV sNS1 secretion efficiency, protein amounts, and oligomeric composition between different mammalian and tick cell lines. They are now investigating the glycosylation and structural diversity of TBFV sNS1 across these systems. At the same time, they are developing experimental tools to determine whether sNS1 is secreted in tick saliva during feeding and how it may influence the vector–host interface. While both sNS1 and tick saliva are known to modulate host immune responses, it remains unknown whether sNS1 is released at the tick bite site. By combining molecular and vector-focused approaches, this work will provide new insight into NS1/sNS1 biology in mammalian hosts and tick vectors and advance our understanding of vector–host interactions that shape TBFV transmission and pathogenesis. No human or animal studies were conducted.

### 2.5. Molecular Foundations of Viral Infections

Annabel Anyang, together with Tyler Starr from the Department of Biochemistry, University of Utah, discussed their work on the molecular evolution of zoonotic coronaviruses in minks. Zoonotic spillover, where pathogens jump from animals to humans, poses a major threat to global health, with emerging coronaviruses responsible for multiple high-impact, cross-species outbreaks over the past two decades. Intermediate host animals have played a pivotal role in these events, acting not only as transmission vessels but also as environments where viruses can acquire adaptive mutations that enhance their zoonotic potential. Therefore, understanding the zoonotic role of intermediate hosts is crucial, since these animals serve as evolutionary bridges between wildlife reservoirs and humans. Farmed minks are of particular concern due to their documented capacity to harbor wildlife coronaviruses at the animal–human interface. Yet, their role, particularly their evolutionary capacity to drive viral adaptation, remains poorly understood. During the pandemic, they served as hosts where SARS-CoV-2 rapidly evolved, raising critical questions about their role in future coronavirus emergence, especially in regions where mink farming intersects with wildlife-rich ecosystems, potentially creating spillover hotspots. This project aims to elucidate the evolutionary mechanisms by which coronaviruses adapt in minks, driving spillover potential. Specifically, their research team aims to define the molecular signatures of entry-related (spike protein) mutations that arise during coronavirus adaptation in minks and determine how these changes contribute to the risk of viral spillover into humans. By integrating high-throughput receptor binding assays, deep mutational scanning, and pseudovirus entry assays, this study will provide a comprehensive understanding of how farmed minks may serve as key evolutionary intermediates driving the emergence of zoonotic coronaviruses. This work will contribute to pandemic preparedness by identifying molecular pathways of host adaptation and highlighting how human activities, such as fur farming, can create evolutionary hotspots that foster future viral spillover, with significant implications for predicting and mitigating future zoonotic threats. No animal or human studies were performed.

Andrew Bubak from the Department of Neurology, University of Colorado Anschutz Medical Campus, discussed his work on the hidden role of VZV reactivation in neurodegenerative diseases and stroke. Varicella Zoster Virus (VZV) infects over 95% of the global population. Following primary infection, it causes chickenpox, after which the virus establishes lifelong latency in neuronal ganglia. Under conditions such as aging or immunosuppressive events (e.g., medications, stress), VZV can reactivate, leading to the painful skin rash known as shingles (zoster). Increasing evidence from large-scale epidemiological studies suggests that VZV reactivation may also play a significant role in the development of severe neurological diseases, including vascular dementia, Alzheimer’s disease (AD), and stroke. However, a major challenge in confirming VZV’s causative role has been the absence of detectable infectious virions in affected tissues. In this study, Andrew and his research team identified a novel mechanism where VZV-induced extracellular vesicles (EVs) contribute to these neurological diseases, even in the absence of infectious virions. Specifically, they demonstrated that non-infectious EVs derived from the plasma of zoster patients can trigger prothrombotic and pro-inflammatory responses months after rash resolution and antiviral treatment. Additionally, EVs from VZV-infected human trigeminal neurons were found to impair the phagocytic activity of macrophages and microglia towards Aβ42, exacerbating amyloid accumulation in AD models. Analysis of EV content revealed a single VZV protein (immediate-early 62) and several miRNAs, which are under investigation as potential causal agents. These findings introduce a novel mechanism by which VZV may contribute to neurologically linked diseases, even without the presence of infectious virions in the affected tissues. No animal or human studies were performed.

Talia J. Byrne-Haber ^1,2^ together with Phillida A. Charley ^1^, Laura A. Pulscher ^1^, Shekinah Johnson ^2^, Arianna Joob ^1^, Tony Schountz ^1^, and Gilbert John ^2^ (^1^ Dept. of Microbiology, Immunology, and Pathology, Center for Vector-Borne Infectious Diseases, Colorado State University, ^2^ College of Veterinary Medicine and Biomedical Sciences, Colorado State University) presented their work on Surveillance and attempted isolation of Sin Nombre Virus in rodents of the American Southwest. The Four Corners region of the American Southwest (Colorado, New Mexico, Arizona, and Utah) is home to free-ranging livestock, wildlife, and the pathogens they host. Of particular concern in this region is Sin Nombre virus (SNV); it is a negative-sense RNA virus in the family *Hantaviridae* with a high fatality rate in humans who develop hantavirus cardiopulmonary syndrome (HCPS). In conjunction with a coronavirus surveillance study of livestock, they trapped and opportunistically tested small rodents for SNV infection. Using an enzyme-linked immunosorbent assay (ELISA) to detect antibodies to SNV nucleocapsid protein, they found that most rodents were seronegative or seropositive with low titers. The original testing on lung tissue homogenate by polymerase chain reaction (PCR) resulted in a few positives for SNV. The few viral sequences the research team did obtain contained polymorphisms, making it difficult for the gene-specific primers to effectively anneal and amplify. Upon designing new primers using the viral sequence positive sample, they found more than 50% of the rodents were infected with SNV, a substantial increase from our original estimates using ELISA, suggesting recent infections. They are currently attempting to isolate these viruses from rodent lung homogenates for further characterization and generation of new hantavirus isolates. All animal studies were performed following guidelines and protocols approved by the Institutional Animal Care and Use Committee of Colorado State University. This research was funded by the National Institutes of Health R21AU163444 and Animal and Plant Health Inspection Service (APHIS) AP23OA000000C020, from the U.S. Department of Agriculture’s Animal and Plant Health Inspection Service.

Michelle Galvan, with Mary Nehring, Treana Mayer, Christie May, Angela Bosco-Lauth, and Sue VandeWoude from Colorado State University’s Department of Microbiology, Immunology, and Pathology, presented her research on feline host susceptibility to Influenza A Virus (IAV), which has historically been understudied. Prior to 2013, felids were considered resistant to IAV, specifically to Avian Influenza Viruses. However, cats are known as susceptible hosts to certain species of Highly Pathogenic Avian Influenza (HPAI), including the current outbreak of H5N1 in North America. Despite this, research on viral-host susceptibility and exposure dynamics is limited. This knowledge gap has become increasingly evident following novel detections of H5N1 in U.S. dairy cattle in March 2024, with fatal cases in domestic cats arising. Given the spillover potential and close proximity between felines and humans, establishing the prevalence of IAV in domestic cats will prove vital to understanding mammalian transmission risk. To investigate this, contemporary and historical domestic cat sera were screened for Influenza A antibodies using a competitive ELISA kit (Innovative Diagnostics). From May to August 2024, 150 serum samples were opportunistically collected from Colorado State University’s Veterinary Diagnostic Laboratory, alongside 200 archived serum samples dated between 2006 and 2015 from six studies across the U.S. and Sydney, Australia. A sample was reported positive with a mean ratio percentage of <45% ((OD_sample_/OD_negative control_) × 100), suspect between 45 and 70%, and negative > 70%. Of the seven experimental groups, each had at least one suspect, with seven total positives for IAV antibodies, with non-negative prevalence between 2.68 and 5.41%. Based on internal validation using specific pathogen-free and experimentally infected cats, this kit can accurately detect IAV antibodies, demonstrating potential IAV exposure as early as 2010 in our field samples. To confirm their results, suspect and positive samples will be followed up with hemagglutination inhibition assays (HAI). Future HAI analysis will help determine the specific HA subtype of IAV these cats have immunity against, helping to provide insight into the evolution of IAV’s dynamics in these peridomestic mammalian hosts. All animal studies were performed following guidelines and protocols approved by the Institutional Animal Care and Use Committee of Colorado State University. Funding was supported by the National Institute of Health T34 MARC Grant Number T34GM140958 and by the USDA NWRC CSU Cooperative agreement: AP23WSNWRC00C107. Contents are the authors’ sole responsibility and do not necessarily represent official USDA or NIH views.

Ava Grunow, with Heather Callaway from Montana State University’s Department of Chemistry and Biochemistry and Department of Microbiology and Cell Biology, discussed her project designing ankyrin repeat proteins to image viral glycoproteins via cryo-electron microscopy. Viral glycoproteins play a central role in infection by mediating host cell entry, making them potential targets for vaccines and antiviral therapeutics. However, their small size, conformational flexibility, and instability outside the viral membrane make them extremely difficult to study. These challenges are especially evident in the rabies virus glycoprotein (RABV-G). Despite its importance, high-resolution structural information on RABV-G remains limited, hindering the development of vaccines and treatments for rabies. Cryo-electron microscopy (cryo-EM) is a widely used technique for determining viral protein structures, but it has limitations when imaging proteins smaller than 50 kDa or those that are highly flexible. This is important because RABV-G is 60 kDa. Designed ankyrin repeat proteins (DARPins) offer a strategy to overcome these challenges. By acting as connector molecules, DARPins can link a protein of interest to a larger, more rigid protein scaffold, increasing the effective molecular size and rigidity needed for high-resolution cryo-EM imaging. This project was divided into three phases: generating DARPin repeats and a library, validating a surface display vector with a GFP-specific DARPin, and developing an imaging scaffold from two plasmids. During phase one, Grunow was able to optimize a protocol to generate the DARPin repeats. In phase two, she determined that a HisTag on the surface display vector causes non-specific binding of the GFP-DARPin interaction, requiring its removal. In phase three, she tested two strategies for scaffold expression and assembly: co-transformation of two plasmids with distinct antibiotic resistances and cloning both scaffold protein sequences onto a single plasmid. The single-plasmid approach produced more promising results, suggesting greater potential for efficient scaffold assembly. This research was funded by Montana INBRE and NIH. No animal or human studies were performed.

Farzana Hossain, Phillida A. Charley, Erika Zhan, Natasha Hodges, Corey Rosenberg, and Tony Shountz (Colorado State University, Department of Microbiology, Immunology, and Pathology) discussed the infection kinetics of sarbecoviruses in a Jamaican Fruit Bat cell line. Regardless of various reports of SARS-CoV-2-related viruses (sarbecoviruses) in horseshoe bats (*Rhinolophus* spp.), research on other sarbecoviruses from bats is limited because few are available for infection studies. Many sarbecoviruses that have similar receptor binding domains (RBD) as SARS-CoV-2 that bind ACE2 have been found and tested against different cell lines. BANAL-52-CoV and BANAL-236-CoV are two sarbecoviruses from horseshoe bats that were found to be among the closest whose RBDs are nearly identical to SARS-CoV-2’s RBD. She has determined that primary kidney epithelial cells (Ajk) from Jamaican fruit bats (*Artibeus jamaicensis*) are not susceptible to sarbecoviruses. A serotype 5 adenovirus vector, Ad5, that encodes human ACE2 (h-ACE2) transduces the cells with h-ACE2. However, no virus replication was detected following SARS-CoV-2 inoculation, suggesting that while the cells are susceptible, they are not permissive for SARS-CoV-2. She tested the susceptibility of these transduced kidney cells to BANAL-52-CoV and BANAL-236-CoV and detected virus replication of both BANAL-CoVs with peaks at 48 h and 72 h post inoculation, respectively. She is expanding their research further by analyzing transcriptomics data of SARS-CoV-2 and BANAL-52-CoV infected cells to determine the replication kinetics of both viruses in human ACE2 bat cells. No animal or human studies were performed.

Austin Knight and Olve Peersen (Colorado State University, Department of Biochemistry and Molecular Biology) presented their studies on how viral RNA polymerase adaptations increase NTP binding for efficient large-genome nidovirus replication. The SARS-CoV-2 virus was responsible for the COVID-19 pandemic and is a member of the large-genome nidoviruses, whose single-stranded RNA genomes range from 28 to 32 kb. These viruses evolved from the ancient picornaviruses, with genomes ranging from 7 to 9 kb. The coronaviral nsp12 and picornaviral 3Dpol RNA-dependent RNA polymerases (RdRPs) replicate their respective genomes, and while the two share an evolutionarily conserved structure found among all viral RdRPs, they bear different active-site geometries. While seemingly minor, these conserved structural differences in the active site have major effects on replication speed and fidelity. Knight engineered the coxsackievirus 3Dpol active site to mimic that of medium- and large-genome viruses’ active sites to investigate the evolutionary role of Glu161, Arg174, and Phe232. By utilizing rapid stopped flow and quenched flow enzyme assays, he discovered increased rates of NTP loading as the Arg174 side chain is destabilized, suggesting that a rigid Arg174 position is a kinetic barrier to NTP active site entry. This adaptation is critical to allowing nsp12 to have a four-fold faster polymerization rate than 3Dpol and is a necessary adaptation to efficiently replicate the significantly larger coronaviral genome while evading the cell’s innate immune response. He also reports that Phe232 limits a post-chemistry event, providing a deeper mechanistic understanding of the viral RdRP catalytic cycle. No animal or human studies were performed.

Shanika Kothalawalage, along with Heather Callaway (Montana State University, Department of Chemistry), discussed her efforts in optimizing the interactions between the rabies virus glycoprotein (RABV-G) and the host p75 neurotrophin receptor (p75NTR) for cryo-EM via kinetic profiling. Rabies virus (RABV) is a neurotropic lyssavirus that causes nearly 59,000 deaths per year. Its ability to infect the nervous system is thought to be mediated by interactions between RABV-G and host receptors, including the p75 neurotrophin receptor. Despite extensive evidence that RABV-G binds to p75NTR, the exact structural details and the key residues responsible for binding have not been fully identified, which presents a critical gap in understanding the virus’s entry process. Her study aims to identify high-affinity p75NTR/RABV-G combinations suitable for cryo-EM structural determination by measuring binding kinetics and identifying key residues involved in the interactions. These can be mutated or combined to further enhance binding. Previous studies show that the cysteine-rich domain 1 (amino acids 32–66) of p75NTR is required and sufficient for RABV-G binding. Kothalawalage performed a sequence alignment over these residues for rabies-susceptible and non-susceptible species and created chimeric p75NTRs with residues unique to these species. Mutants generated were functionally validated through binding kinetics using Bio-Layer Interferometry (BLI) to identify residues essential for interaction with RABV-G. Mutations H39D and H39T disrupted binding, while protonation of H39 at pH 6.0 slightly improved binding, confirming the functional importance of H39D residue in p75NTR-RABV-G interaction. They are continuing to search for variants with sufficiently enhanced binding affinity to serve as potential candidates for cryo-EM structural analysis. These structures will reveal key binding residues and advance our understanding of viral entry mechanisms, guiding future therapeutic strategies. Research was supported by the National Institute of General Medical Sciences of the National Institutes of Health under Award Number P20GM103474. No animal or human studies were performed.

Siddarth Krishnamurthy from the University of Colorado Anschutz Medical Center’s Department of Immunology and Microbiology presented the work of his team, consisting of Juan Samaniego Castaneda, Holly Vose, Amonrat O’Brien, Brian Wasik, Claudia Rivera, Yasmine Belkaid, Colin Parrish, and Susan Baker. Krishnamurthy’s talk revolved around the nutritional and genetic synergy that determines colonic tropism in enteric coronaviruses. Many zoonotic viruses, like coronaviruses, infect the gastrointestinal tract of their reservoir host. Yet, when they emerge in humans, they are respiratory viruses. These environments differ both in their microbiota and immune landscape, yet these viruses productively infect both compartments. Therefore, he aimed to understand how coronaviruses adapt to initiate intestinal infection. He developed a novel intestinal coronavirus infection model using the murine coronavirus, mouse hepatitis virus Y (MHV-Y), that naturally infects the intestinal tract of mice. This tropism contrasts starkly with that of its more commonly studied related strains, MHV-A59 and MHV-JHM, which are hepatotropic or neurotropic. These differences in tropisms manifest despite these strains using the same entry receptor, mouse CEACAM1. They found that infection of the colon, but not mesenteric lymph node (mLN) and Peyer’s patches, required butyrate derived from microbiota fermentation of dietary fiber. Comparative genomics between enteric versus non-enteric strains of MHV revealed that non-enteric strains consistently lacked the envelope lectin, hemagglutinin esterase (HE). This envelope lectin binds 4-O acetylated sialic acid (4AcSA), which they found to be highly abundant on the enteric glia in the colonic lamina propria. They developed a reverse genetic system for MHV-Y and found that HE-deficient MHV-Y (MHV-YΔHE) could not infect the colon, while maintaining infection in the Peyer’s patches and mesenteric lymph node. Conditions that maintained colonic intestinal infection competence, such as a high-fiber diet and wild-type viral genetics, correlated with a dramatic reduction in highly differentiated antimicrobial-producing goblet cells. Transmission to naïve mice required colonic infection, as conditions that lacked colonic infection, such as a low-fiber diet or MHV-YΔHE, did not permit transmission to co-housed mice. These results show that both attachment proteins separate from the entry receptor (spike) and environmental cues dictate coronavirus tropism. All animal studies were performed following guidelines and protocols approved by the Institutional Animal Care and Use Committee of the University of Colorado at Anschutz Medical Center, Protocol #01477 IBC #1705.

Leonard W. Ma ^1^, along with David A. Price ^1^, Shane Miersch ^3^, Divya Mehta ^2^, Sachdev S. Sidhu ^3^, Gaya K. Amarasinghe ^2^, and Daisy W. Leung ^1^ (^1^ Department of Medicine, Washington University School of Medicine, St. Louis, MO 63110, USA, ^2^ Department of Pathology and Immunology, Washington University School of Medicine, St. Louis, MO 63110, USA, ^3^ Anvil Institute, University of Waterloo, Waterloo, ON, Canada) presented their efforts to develop molecular tools in order to characterize interactions between Marburg VP40 and the host actin cytoskeleton. Marburg virus is an enveloped negative-sense single-stranded RNA filovirus that causes rare but deadly outbreaks of Marburg virus disease (MVD) in humans, the most recent in Tanzania in early 2025. Marburg virus encodes only seven structural proteins; therefore, exploitation of host factors is necessary for efficient viral replication. Notably, Marburg virus subverts the actin cytoskeleton during viral entry, budding, egress, and spread, likely through the major matrix protein VP40. Marburg VP40 (mVP40) can self-assemble into higher-order oligomers to form VLPs and is crucial in organizing viral assembly and budding, thus linking the viral nucleocapsid with the envelope. Previous studies have shown co-localization and interaction between mVP40 and actin filaments. However, it is poorly understood how these interactions coordinate viral assembly and egress, as well as the underlying molecular mechanisms. Ma described the development of tools to facilitate the characterization of protein–protein interactions involving mVP40. Synthetic antibodies generated by phage display technology were validated for binding to purified recombinant mVP40 protein using in vitro biochemical and biophysical methods. Further characterization will be performed in structural and cell biological studies. Overall, this work provides the basis for additional studies to evaluate interactions between Marburg VP40 and the host actin cytoskeleton that can provide mechanistic insight and define potential targets for therapeutic development. No animal or human studies were performed.

Lauren Malsick, in conjunction with Amanda Bartels, Paris Kiehl, Sophie Kiehl, John Anderson, E. Handly Mayton, Brooke Enney, Hunter Ogg, Corey L. Campbell, and Brian Geiss from Colorado State University’s Department of Microbiology, Immunology, and Pathology, presented their research on investigating subgenomic flaviviral RNAs (sfRNAs) and alternative splicing in Jamaican fruit bat cells. Flaviviruses, including mosquito-borne pathogens such as dengue (DENV), West Nile (WNV), and Zika (ZIKV), cause significant morbidity and mortality across the globe. It has been determined that many species of bats can be infected with flaviviruses, but these bats typically do not develop severe disease. Why bats are infected by flaviviruses but do not get the disease is unclear. Bats are vectors for several human viral pathogens, and therefore it is important to clarify their role in viral transmission cycles between different species and their potential as reservoirs for future spillover events. Subgenomic flaviviral RNAs (sfRNAs), a key pathogenesis determinant for flaviviruses in mammalian species, have been detected in bats, but it is currently unknown if sfRNAs affect gene expression in bats as in other species. In this study, Malsick found that WNV productively infects primary *Artibeus jamaicensis* bat cells but shows minimal cytopathic effect, which is strikingly different than other infectable mammalian cell types. To further understand this phenotype, she examined how sfRNAs affect innate immune responses in bat cells, including characterization of sfRNA species in bat cells, how disruption of sfRNA structures alters viral replication, and how sfRNA disruption affects host innate immune response gene expression via RNA-Seq analysis. She has observed that sfRNA-defective WNV significantly alters innate immune gene expression patterns during infection. Additionally, she was able to determine that sfRNAs in bat cells contribute to alternative splicing with an exon skipping phenotype, validating previous literature in other model organisms. Overall, this work validates that WNV can replicate well in bat cells, indicating that *Artibeus jamaicensis* could serve as a reservoir species for WNV and potentially other flaviviruses. No animal or human studies were performed.

Emerson Ross, along with Grace Campagnola and Olve Peersen from Colorado State University’s Department of Biochemistry and Molecular Biology, shared his research regarding substrate-induced pH dependence of peptide cleavage by poliovirus 3C protease. The picornaviral 3C protease is a critical enzyme responsible for processing the viral polyprotein, which is an essential step for viral replication. 3C protease activity is known to be sensitive to pH due to the classic protease catalytic triad active site, but a recent study has also suggested substrate interactions can affect active site protonation states. To address this, he examined the pH-dependent cleavage profiles of 3C protease using multiple peptide substrates to determine whether variations in activity were influenced by substrate sequence. Cleavage rates were measured across a range of pH conditions using a fluorescence polarization-based protease assay. Analysis of the fluorescence polarization data revealed that a two-phase activity model better described substrate cleavage for all peptides compared to a single-phase model. While the overall activity was strongly pH-dependent, the differences among substrates were minimal, indicating that the pH profile of 3C protease is not significantly affected by substrate interactions and is instead a core catalytic property of the enzyme. This research was funded by the National Institute of Allergy and Infectious Diseases, project number R37AI059130. No animal or human studies were performed.

Nimasha Samarasinghe, with Heather M. Callaway from Montana State University’s Department of Microbiology and Cell Biology, offered new insights on how unique antibody features underlie potent rabies virus neutralization in bats. Bats are a highly diverse group of mammals that harbor numerous viruses, including many that are deadly in humans but may not cause disease in bats. Rabies is one such virus, nearly 100% lethal in humans and responsible for an estimated 40,000–70,000 deaths annually, yet it is comparatively less lethal in bats. She hypothesizes that bat immunoglobulins have unique characteristics that reduce the lethality of rabies. Recent studies have shown that some bat species carry two complete, functional, and distinct immunoglobulin heavy chain loci in their germline, resulting in an expanded antibody repertoire. In addition, elevated temperatures corresponding to flight have shown enhanced antibody binding to the rabies surface glycoprotein, RABV-G. Here, Samarasinghe immunized Jamaican fruit bats with a killed, veterinary rabies vaccine and collected serum samples. These samples were subjected to ELISA and virus neutralization assays to measure the total rabies-specific antibodies and their capacity to neutralize the virus. Her results show that bats produce potently neutralizing antibodies against the rabies virus with a notable individual and sex-based variation in total and neutralizing antibodies. Interestingly, male bats produced higher levels of both total and neutralizing antibodies compared to females. These results provide a start to understanding the unique properties in certain bat rabies-specific antibodies that may underlie their enhanced neutralizing potency, offering valuable insights to develop future rabies therapeutics. All animal studies were performed following guidelines and protocols approved by the Institutional Animal Care and Use Committee of Montana State University. This research was funded through internal funds from Montana State University.

Laura A. St Clair, with Jennifer A. Liu, Sabal Chaulagain, Joseph P. Hoffmann, Brittany Siebert, Sneha Mahesh, John S. Lee, Emily G. Watters, and Sabra L. Klein from the W. Harry Feinstone Department of Molecular Microbiology and Immunology at Johns Hopkins Bloomberg School of Public Health, presented her research on how metabolic flipping in B cells drives sex differences in influenza vaccine-induced immunity. Biological sex impacts the effectiveness of vaccines that protect against viruses of pandemic potential, including influenza A viruses. Following influenza vaccination, reproductive-age (18–49 years) females produce greater antiviral antibody responses than age-matched males, which correlates with greater estradiol (E2) concentrations. How E2 drives greater B cell activation is not known, but she hypothesizes that E2 signaling may regulate immunometabolic pathways related to B cell maturation, including mTOR activation, which is indispensable for antibody production. To test this hypothesis, St Clair conducted untargeted metabolomics analysis of splenic B cells isolated at 7 days post-boost (DPB) from young adult male and female mice vaccinated with either a mock or H1N1 vaccine. She observed divergent B cell metabolic phenotypes in vaccinated animals, with notable elevations in pathways involved in lipid and protein biosynthesis in females compared with higher glycolytic activity in males. To evaluate whether these differences were due to differential mTOR activation, she analyzed splenic B cell protein expression in a separate cohort of mice via Western blot. She observed that B cells from males and females had differential expression of mTOR and metabolism-related proteins at resting state (i.e., unvaccinated) versus activated state (i.e., post-vaccination). At the resting state, males had greater expression of metabolic proteins than females, but these phenotypes reversed post-vaccination. Rapamycin (mTOR inhibitor) treatment abrogated sex differences in vaccine-induced immunity and protection against live virus challenge. Additionally, sex differences in influenza vaccine-induced immunity are lost with age, with a more pronounced decline in aged females, which correlates with a decline in circulating E2. She also shows that sex differences in metabolic protein expression are lost with age in vaccinated aged (17-month-old) mice. Taken together, these data suggest that E2-mediated regulation of mTOR in B cells is a possible mechanism governing sex differences in vaccine-induced immunity. All animal studies were performed following guidelines and protocols approved by the Institutional Animal Care and Use Committee of Johns Hopkins University.

Kayla M. Weldon, in conjunction with Kolya Isterabadi, Holly A.F. Stessman, and Michael Belshan from the Department of Medical Microbiology and Immunology at Creighton University School of Medicine, presented her research on how annexin A5 regulates HIV-1 infection. Like other viruses, Human Immunodeficiency Virus (HIV) is an obligate intracellular parasite that requires host cell factors and machinery for productive replication. Identifying novel pathways and mechanisms that regulate HIV infection may unlock new strategies to inhibit virus replication and impair or eliminate HIV persistence in people living with HIV. In these studies, CRISPR-Cas9-treated Jurkat T cells with altered susceptibility to HIV-1 infection were cloned by limiting dilution. RNA was isolated from seven Jurkat T-cell lines with substantial differences in susceptibility (3 high and 4 low vs. parental cell line) and analyzed by total RNA sequencing and analysis. Differentially expressed genes were identified by an adjusted *p*-value of <0.001 and log2 change of >1.0. One hundred and five genes were independently assessed by qRT-PCR. Several members of the Annexin (Anx) family were found to have altered expression among the cell lines. Annexins (Anxs) are a highly conserved family of proteins with identified roles at various stages in the life cycles of several viruses, including interacting with viral envelope proteins, promoting membrane fusion, and subsequent entry of the viral genome into the host cell. Several Anxs were investigated for their role in HIV infection through over-expression and knock-down studies. Among those, stable knockdown of Annexin A5 (AnxA5) decreased susceptibility to HIV-1 during single-cycle infection assays. Knockdown of AnxA5 also decreased virus production, and the infectivity of the virus produced was reduced compared to the wild-type virus. These data indicate that AnxA5 regulates HIV-1 replication. Ongoing studies are examining the molecular mechanism of AnxA5 on HIV-1 replication. No animal or human studies were performed.

Jamin Willoughby Jr., with Tyler N. Starr from the Department of Biochemistry at the University of Utah, presented his research on deep mutational scanning of VRC01 HIV broadly neutralizing antibody reveals potential pathways of HIV Env N276 glycan accommodation. Despite decades of intense research, no established cure or vaccine for Human Immunodeficiency Virus (HIV) has been developed. One reason for the lack of an HIV vaccine is the high rate of HIV evolution and the heavy glycosylation of the viral envelope (Env) protein, which is the only known target of broadly neutralizing antibodies (bnAbs). bnAbs that target the CD4 binding site (CD4bs) on Env are promising templates for an HIV vaccine, but current HIV immunogens are thus far unable to raise antibodies that neutralize diverse HIV strains. The lack of neutralization potency and breadth amongst many CD4bs bnAbs is often due to a specific Env glycosylation site at asparagine 276. This N276 glycan is present in ~95% of HIV strains. While the broadest and most potent CD4bs bnAbs can accommodate the N276 glycan, few evolutionary routes of N276 glycan accommodation are known. Using his platform of deep mutational scanning leveraging yeast surface display, he assessed how all possible single amino acid mutations in the variable domains of the CD4bs bnAb VRC01 and its naïve precursor affect binding affinity to 45_01dG5, a native Env protein that lacks the N276 glycan, and 45_01dH1, a closely related N276 glycan-containing Env strain. Willoughby Jr. reveals both known and previously unseen affinity-enhancing mutations to 45_01dG5 and 45_01dH1 in VRC01. Additionally, he saw mutations in the VRC01 naïve precursor that increase affinity to 45_01dH1 to various degrees. The results herein provide a baseline of the full flexibilities and constraints of VRC01 accommodation of the N276 glycan barrier, allowing insight into the evolutionary pathways that guide increasing the breadth of these CD4bs bnAbs. Understanding the broader sequence–function relationship of CD4bs bnAbs will allow for a deeper insight into how these antibodies can overcome the N276 glycan, directly informing HIV vaccine immunogen design. This research was funded by NIH/NIAID DP2AI177890. No animal or human studies were performed.

## Data Availability

No new data were created or analyzed in this study. Data sharing is not applicable to this article.

